# Self-balanced switched capacitors based thirteen level three-fold multilevel inverter for solar PV applications

**DOI:** 10.1038/s41598-025-21760-6

**Published:** 2025-10-29

**Authors:** Niraj Kumar Dewangan, Kasinath Jena, Tarun Kumar Tailor, Devesh Umesh Sarkar, Murli Manohar

**Affiliations:** 1 Department of Mechatronics, Manipal Institute of Technology, Manipal Academy of Higher Education, Manipal, 576104 India; 2 Department of Electrical and Electronics Engineering, ARKA JAIN University, Jharkhand, India; 3 Electrical Engineering Department, Institute of Technology, Nirma University, Ahmedabad, Gujarat India; 4 Department of Electrical Engineering, Indian Institute of Technology Palakkad, Kanjikode, India; 5 Centre for Internet of Things, Madhav Institute of Technology & Science, Madhya Pradesh, Gwalior India

**Keywords:** Cost function, Multilevel inverter topologies, Switched capacitors, TSV, Electrical and electronic engineering, Electronic and spintronic devices, Superconducting devices

## Abstract

While the conventional topologies of multilevel inverters (MLIs) operate with unity voltage gain, switched capacitors-based MLIs (SCMLIs) offer a solution to realize an inherent voltage gain of more than one, thereby stepping up the voltage in the process of DC to AC conversion. This work proposes a novel SCMLI constituting thirteen levels, requiring only one DC input and 3-capacitors to achieve an inclusive three-fold gain in voltage. The number of power switches employed in the proposed module is thirteen, where nine switches peak-inverse-voltage (PIV) is considered as one-third of the output voltage amplitude. Experimental results validate the proposed inverter (PI), demonstrating its efficacy. The superior performance of the PI with respect to component count, power switch voltage ratings, and cost function (CF) is highlighted by comparison with other SCMLIs. The total standing voltage (TSV) per level, expressed in per unit with respect to ${V}_{in}$ is 1.307, while the PIV per level is 0.153, which is competitive with recent literature. Additionally, the CF is 4.538, lower than other designs, enhancing the performance of the structure for real-time applications.

##  Introduction

Many applications favor multilevel inverters (MLIs) over two-level inverters due to numerous advantages. Power switches with a PIV than the multilevel AC output operating voltage increase efficiency and reliability. Due to their improved harmonic profile, MLIs require less filtering, simplifying system design, and saving costs. Reduced dv/dt stress on the load extends its lifespan and improves reliability. Fault-tolerant MLIs are more resilient in crucial applications. Neutral point clamped (NPC), flying capacitors (FC), and cascaded H-bridge (CHB) MLI topologies are used in renewable energy systems, electric motors, and vehicle electrification. MLIs improve voltage quality, electromagnetic interference, and operational flexibility for these applications. As a result, MLIs continue to advance power electronics and meet the growing demand for reliable and effective energy conversion in modern technological systems^1–3^. These classical topologies operate with a gain of unity, and hence, there is no inherent stepping up of voltage. In the past few years, a new family of MLIs has emerged with the capability of operating with a voltage gain of more than unity. These structures use switched capacitors (SC) to step up the voltage magnitude and are generally referred to as SCMLIs^4^. Inductor-less configuration and self-balancing of capacitors are other attractive features of SCMLIs^5^.

As a result of their various advantages, numerous SCMLI topologies have recently evolved^4–17^. In^4^, the authors have proposed a switched-capacitors module with minimal switch count and TSV. Still, it is incapable of synthesizing a bipolar waveform. Hence, multiple modules are to be used in a cross-connected fashion to realize an alternating waveform, leading to the requirement of multiple isolated DC sources. The 13-level inverter proposed in^5^ operates with a gain of 6. However, it consists of PIV switches equal to the operating voltage. The SCMLI presented in^6^ needs switches with a PIV equal to the input source voltage; however, its component count rises significantly as the number of levels increases. Barzegarkhoo et al.^7^ have presented an easily extendable SCMLI structure using low voltage bearing power switches. Still, the switches are a mix of unidirectional and bidirectional configurations, thereby limiting the modularity. The single-stage SCMLI module proposed by S.S. Lee^8^ is extremely advantageous in terms of PIV ratings of switches, but the overall component count is high. Similarly, highly modular topologies are proposed in^9,10^, but they involve two-stage conversion with the help of four H-bridge power switches, capable of handling the operating voltage. An innovative approach that uses traditional H-bridges and switched capacitors is proposed in^11^ with some additional power switches. However, the capacitor voltage balancing requires a complex methodology in it. A different cascaded module approach for SCMLI is shown in^12^. However, it is limited in its applicability to high-voltage applications since each module requires two power switches to be rated at operational voltage. A different approach modular strategy that has been proposed^13^ includes several components at each level. The generalized structure of single-phase SCMLIs presented in^14^ requires fewer switching devices. Still, the involvement of a two-stage operation requires the use of H-bridge switches having PIV, which is the same as the operating voltage. The topology proposed by He and Cheng^15^ utilizes flying capacitors clamping for the SCMLI in order to attain a large gain, but the approach leads to a high number of elements per level. Highly modular and low PIV topologies^16,17^ provide advantages in some applications.

The topology described in^8^ incorporates switches with a low PIV rating. However, it has a limited voltage gain and requires a relatively high number of switches. A similar trend can be observed in the configurations presented in^18^. Research studies from^19–24^ delve into single-stage, 13-level SC-MLI designs. The converter introduced in^19^ offers moderate voltage gain and a reduced switch count, but its TSV is comparatively higher. Moreover, the number of capacitors required to increase with the rise in load power factor. SC-MLI designs with higher voltage gain have been explored in^20,21^, and^22–24^. The approach in^20^ requires more components, while the design in^21^ depends on switches with higher PIV ratings. The converter discussed in^24^ utilized a higher quantity of switches among these designs. On the other hand, the configurations presented in^22^ and^23^ require capacitors with higher voltage ratings, which add to the overall cost of the system. Additionally, the designs in^20^ and^21^ face challenges with charge balancing across capacitors, especially at lower modulation indices. The SC-MLI design presented in^25^ attains a gain of 3 but is characterized by a relatively large switch count. This configuration also exhibits a high-cost function (CF), requires switches with significant PIV ratings, and shows elevated TSV levels. However, its voltage balancing capabilities deteriorate when operating at lower modulation indices. Furthermore, the asymmetrical topology proposed in^26^ integrates power devices with elevated PIV and TSV ratings, making voltage balancing in this design particularly challenging. These advantages come with drawbacks, such as poor voltage gain and high semiconductor demand, which can reduce efficiency and practicality.

References^27,28^ present common-ground SC based five-level inverter topologies. These configurations enable efficient high-voltage gain while minimizing the part count and suppressing leakage current, making them highly suitable for compact and low-loss power conversion systems. Additionally, the work presented in^29^ introduces a nine-level single-phase SC inverter capable of achieving 4-X gain. This design further enhances performance by reducing the total number of components, minimizing TSV across the switches, and ensuring cost-effective implementation.

Given these factors, SCMLIs with high-resolution output, low PIV switches, high voltage gain, and minimal semiconductor utilization have enormous potential. SCMLIs could become more adaptable and efficient for a broader range of power electronics applications with such improvements. The presented study unveils a single-stage SCMLI module, highlighting the following attributes: (a) a voltage gain of three; (b) generation of a thirteen-level waveform through a single DC source, three capacitors, and thirteen power switches; (c) inherent self-balancing of all capacitors; and (d) ensuring the PIVs of the switches remain substantially lower compared to the operating voltage.

The paper is structured as follows: Section II describes the proposed module structure and operation. The same section also compares it with other topologies mentioned in^4–26^. Section III describes the switching process used in the suggested module. Section IV presents the experimental findings, and section V discusses the conclusions.

## The proposed 13-level inverter and it’s working

**Fig. 1:**
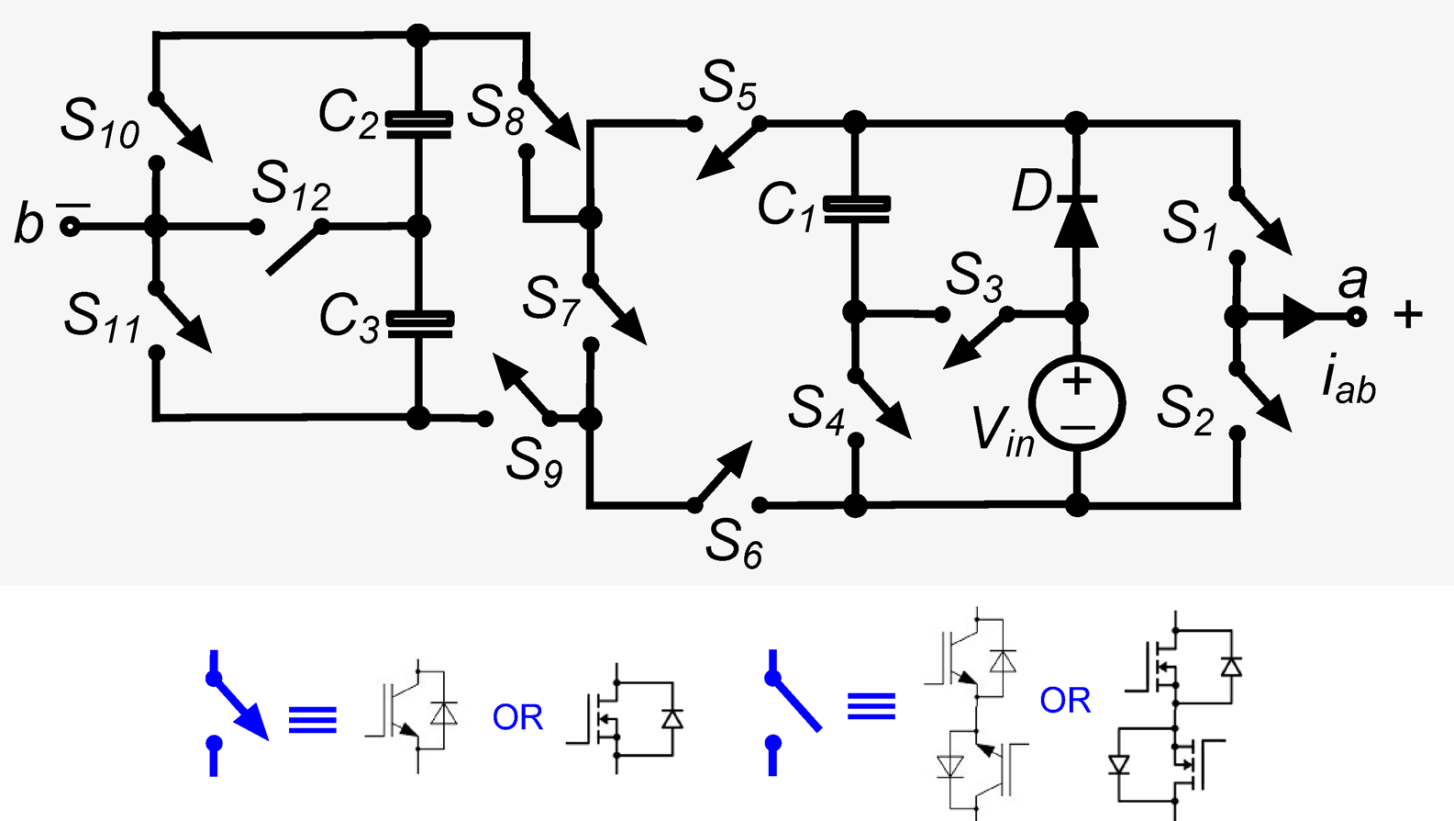
The proposed 13-level inverter.

Figure 1 shows the power circuit diagram of the thirteen-level inverter suggested in this work. It comprises twelve power switches $,$ a diode ‘$\text{D}$’, capacitors (${\text{C}}_{1}, {\text{C}}_{2},\text{ and }{\text{C}}_{3}$), and one DC source (shown with voltage ${\text{V}}_{\text{in}}$). Of the twelve switches, the power switch ${\text{S}}_{12}$ is required to be of bidirectional-blocking-bidirectional-conducting type, while rest of the switches are of unidirectional-blocking-bidirectional-conducting type. The load voltage is represented as ‘${\text{v}}_{\text{ab}}$’. The capacitor ${\text{C}}_{1}$ is to be kept at voltage equal to ${\text{V}}_{\text{in}}$, while the capacitors ${\text{C}}_{2}$ and ${\text{C}}_{3}$ are to be maintained at $0.5{\text{V}}_{\text{in}}$ each. The structure is capable of synthesizing thirteen voltage levels (viz. $\pm 0.5{\text{V}}_{\text{in}}$, $\pm {\text{V}}_{\text{in}}$, $\pm 1.5{\text{V}}_{\text{in}}$, $\pm 2{\text{V}}_{\text{in}}$, $\pm 2.5{\text{V}}_{\text{in}}$, $\pm 3{\text{V}}_{\text{in}}$ and $0$) at the load terminals. The PIV rating of the switches used in the designed inverter is tabulated in Table 1.

**Table 1:** PIV rating of the switches.

PIV	Power switches
$0.5{V}_{in}$	${\text{S}}_{12}$
${V}_{in}$	${\text{S}}_{3}, {\text{ S}}_{4}, {\text{ S}}_{7}, {\text{ S}}_{8}, {\text{ S}}_{9}, {\text{ S}}_{10}, {\text{ S}}_{11},\text{D},$
$2{V}_{in}$	${\text{S}}_{1}, {\text{S}}_{2}, {\text{S}}_{5}, {\text{S}}_{6}$

**Fig. 2:**
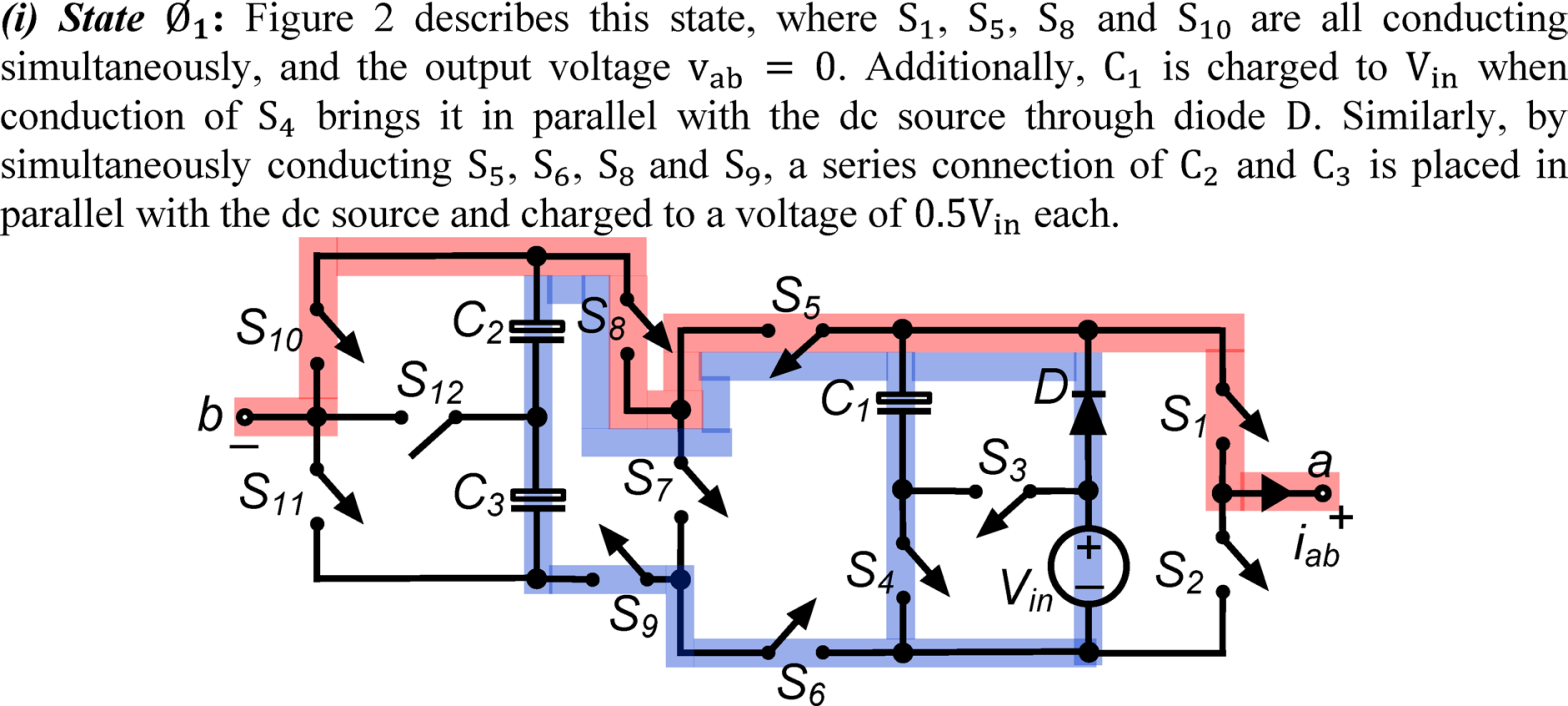
Working state ${\text{\O }}_{1}$ of the PI [${\text{v}}_{\text{ab}}=0$].

The working principle of the PI can be comprehended with the description of fourteen states ${\text{\O }}_{\text{k}} \{\text{k}\hspace{0.17em}=\hspace{0.17em}1\text{ to }14\}$. Each state is a distinct switching combination to synthesize the desired voltage level at the load terminals. Moreover, the capacitors are placed parallel to the input supply so that the voltage across them can be maintained at its desired voltage levels. Consequently, for each state, the route for generating the output voltage is indicated by a bold red line, while a narrow blue line represents the charging pathway for the capacitor(s). These states are elaborated as follows:

*(i) State* ${\O }_{1}$: Fig. 2 describes this state, where ${\text{S}}_{1}$, ${\text{S}}_{5}$, ${\text{S}}_{8}$ and ${\text{S}}_{10}$ are all conducting simultaneously, and the output voltage ${\text{v}}_{\text{ab}}\hspace{0.17em}=\hspace{0.17em}0$. Additionally, ${\text{C}}_{1}$ is charged to ${\text{V}}_{\text{in}}$ when conduction of ${\text{S}}_{4}$ brings it in parallel with the dc source through diode $\text{D}$. Similarly, by simultaneously conducting ${\text{S}}_{5}$, ${\text{S}}_{6}$, ${\text{S}}_{8}$ and ${\text{S}}_{9}$, a series connection of ${\text{C}}_{2}$ and ${\text{C}}_{3}$ is placed in parallel with the dc source and charged to a voltage of $0.5{\text{V}}_{\text{in}}$ each.

**Fig. 3:**
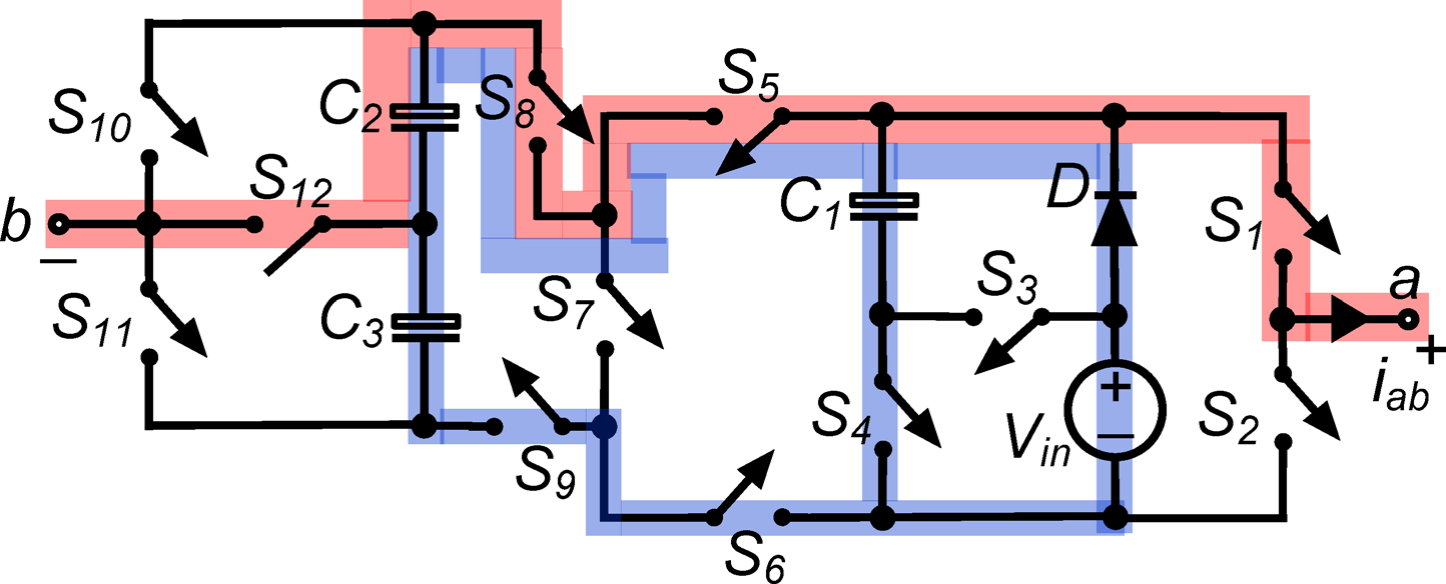
Working state ${\text{\O }}_{2}$ of the PI [${\text{v}}_{\text{ab}}=0.5{\text{V}}_{\text{in}}$].

*(ii) State *${\O }_{2}$*:* Fig. 3 shows switches ${\text{S}}_{1}$, ${\text{S}}_{5}$, ${\text{S}}_{8}$ and ${\text{S}}_{12}$ connecting load terminals to capacitor ${\text{C}}_{2}$consequently, the output voltage ${\text{v}}_{\text{ab}}\hspace{0.17em}=\hspace{0.17em}{\text{v}}_{\text{C}2}=0.5{\text{V}}_{\text{in}}$. Moreover, switch ${\text{S}}_{4}$ conducts capacitor ${\text{C}}_{1}$ in parallel with the dc source via diode $\text{D}$, charging it to ${\text{V}}_{\text{in}}$. In parallel with the dc source, switches ${\text{S}}_{5}$, ${\text{S}}_{6}$, ${\text{S}}_{8}$ and ${\text{S}}_{9}$ link capacitors ${\text{C}}_{2}$ and ${\text{C}}_{3}$ in series. The abovementioned arrangement charges capacitors ${\text{C}}_{2}$ and ${\text{C}}_{3}$ to $0.5{\text{V}}_{\text{in}}$. To run the inverter system efficiently, this state regulates capacitor charging and discharging, optimizing voltage levels and maintaining the desired output.

**Fig. 4:**
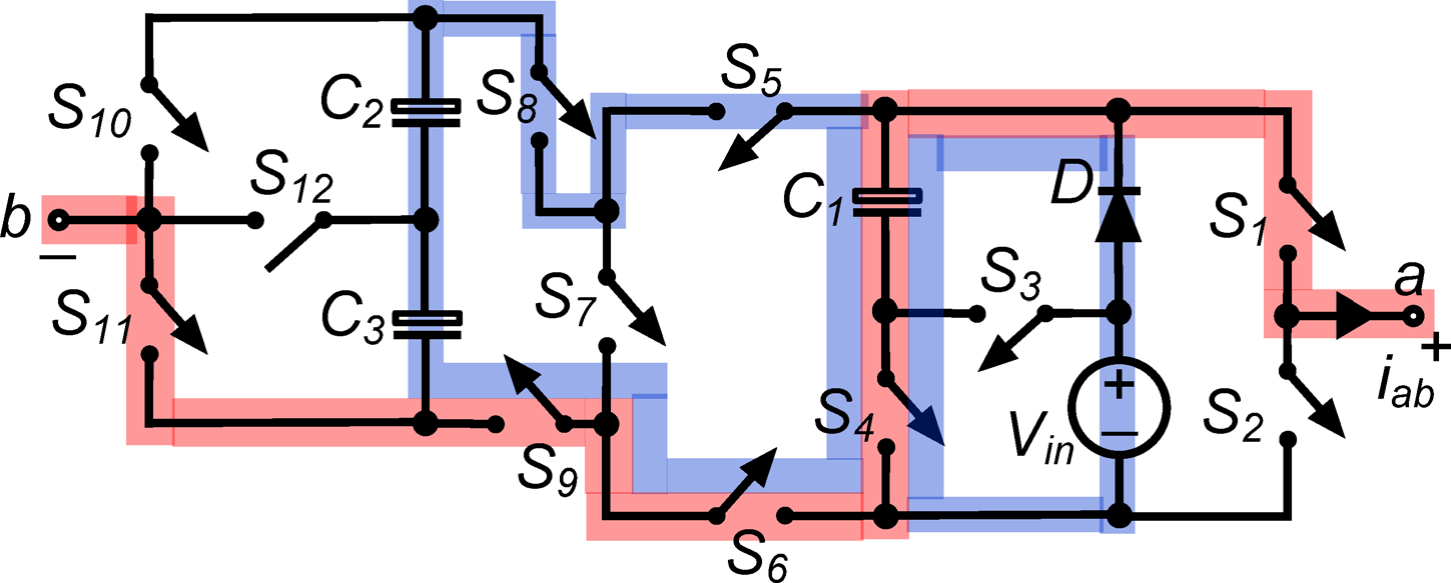
Working state ${\text{\O }}_{3}$ of the PI [${\text{v}}_{\text{ab}}={\text{V}}_{\text{in}}$].

*(iii) State* ${\O }_{3}$ : Fig. 4 shows switches ${\text{S}}_{1}, {\text{S}}_{4}, {\text{S}}_{6}, {\text{S}}_{9}$ and ${\text{S}}_{11}$ connecting load terminals to capacitor ${\text{C}}_{1}$. The output voltage: voltage ${\text{v}}_{\text{ab}}\hspace{0.17em}=\hspace{0.17em}{\text{v}}_{\text{C}1}={\text{V}}_{\text{in}}$, supplying the whole input voltage to the load. Moreover, switch ${\text{S}}_{4}$ conducts capacitor ${\text{C}}_{1}$ via diode $\text{D}$, maintaining its value to ${\text{V}}_{\text{in}}$. Parallel to the dc supply, switches ${\text{S}}_{5}, {\text{S}}_{6}, {\text{S}}_{8}$ and ${\text{S}}_{9}$ connect capacitors ${\text{C}}_{2}$ and ${\text{C}}_{3}$ in series. This setup charges capacitors ${\text{C}}_{2}$ and ${\text{C}}_{3}$ to $0.5{\text{V}}_{\text{in}}$. Maintaining capacitor voltage levels and inverter energy flow in this stage ensures efficient operation and appropriate output.

**Fig. 5:**
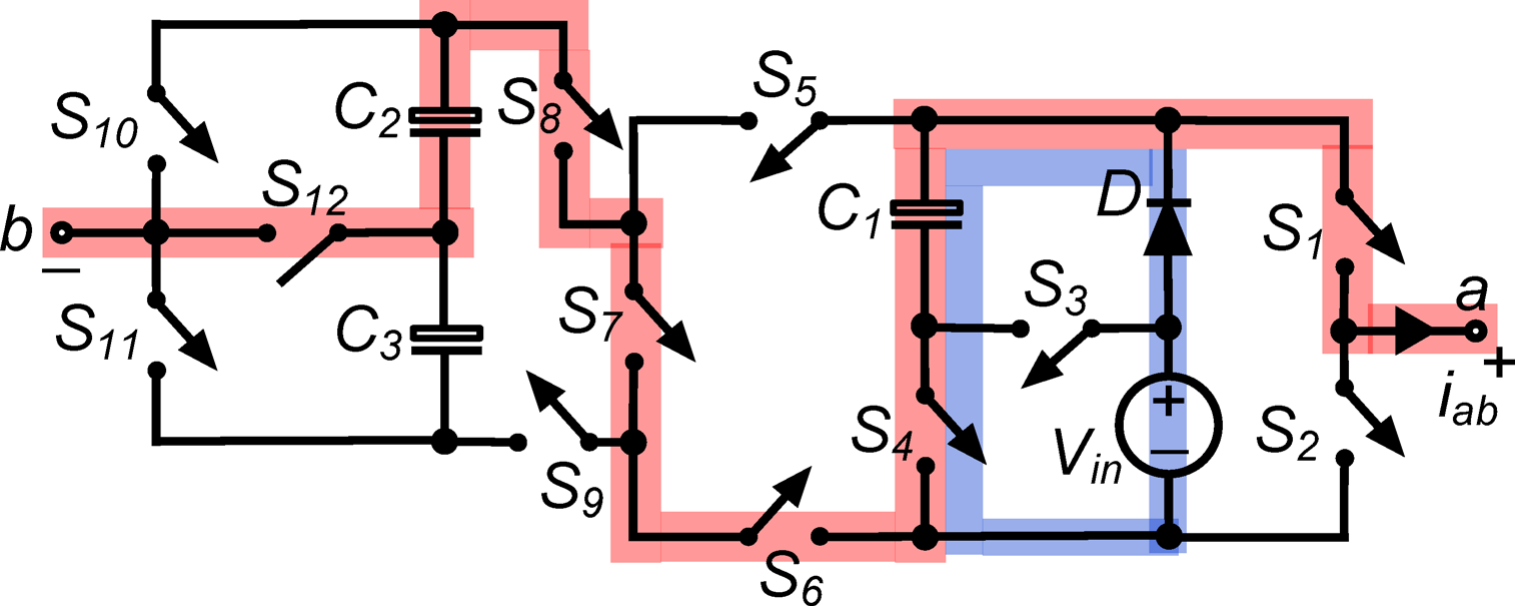
Working state Ø_4_ of the PI [v_ab_ = 1.5V_in_].

*(iv) State* ${\O }_{4}$: In the state depicted in Fig. 5, the load terminals are connected to ${\text{C}}_{1}$ and ${\text{C}}_{2}$ through the simultaneous conduction of ${\text{S}}_{1}, {\text{S}}_{4}, {\text{S}}_{6}, {\text{S}}_{7}, {\text{S}}_{8}$ and ${\text{S}}_{12}$. So, the output voltage ${\text{v}}_{\text{ab}}= {\text{v}}_{\text{C}1}+{\text{v}}_{\text{C}2}=1.5{\text{V}}_{\text{in}}$. Moreover, the conduction of ${\text{S}}_{4}$ places ${\text{C}}_{1}$ in parallel with the dc source via diode $\text{D}$, thereby charging it to ${\text{V}}_{\text{in}}$.

**Fig. 6:**
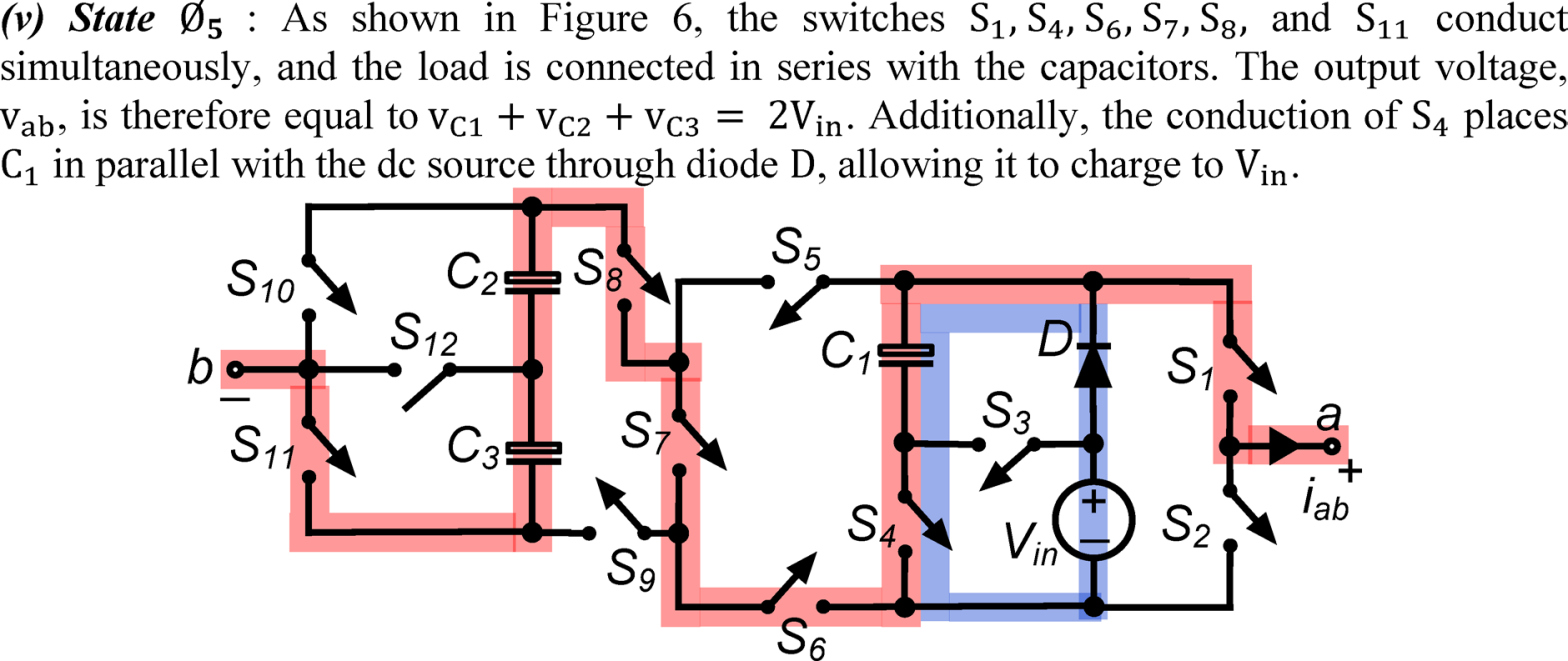
Working state ${\text{\O }}_{5}$ of the PI [${\text{v}}_{\text{ab}}= 2{\text{V}}_{\text{in}}$].

*(v) State* ${\O }_{5}$ : As shown in Fig. 6, the switches ${\text{S}}_{1}, {\text{S}}_{4}, {\text{S}}_{6}, {\text{S}}_{7}, {\text{S}}_{8},$ and ${\text{S}}_{11}$ conduct simultaneously, and the load is connected in series with the capacitors. The output voltage, ${\text{v}}_{\text{ab}}$, is therefore equal to ${\text{v}}_{\text{C}1}+ {\text{v}}_{\text{C}2}+ {\text{v}}_{\text{C}3}= 2{\text{V}}_{\text{in}}$. Additionally, the conduction of ${\text{S}}_{4}$ places ${\text{C}}_{1}$ in parallel with the dc source through diode $\text{D}$, allowing it to charge to ${\text{V}}_{\text{in}}$.

**Fig. 7:**
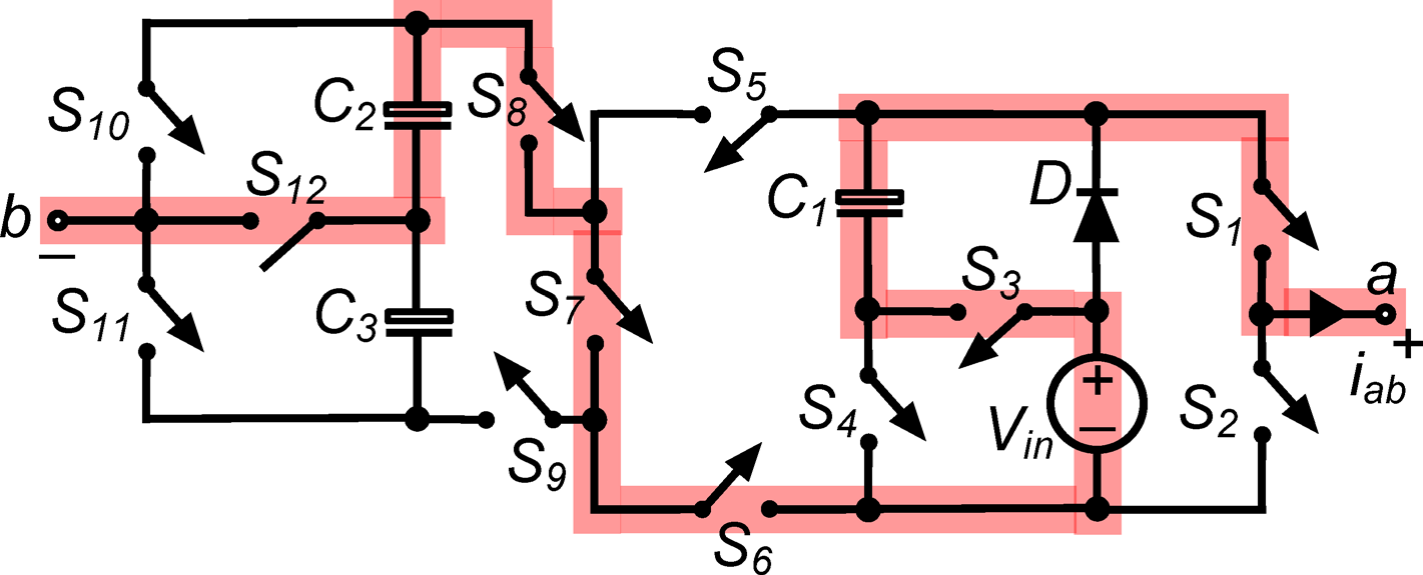
Working state ${\text{\O }}_{6}$ of the PI [${\text{v}}_{\text{ab}}=2.5{\text{V}}_{\text{in}}$].

*(vi) State* ${\O }_{6}$ : The load terminals in this condition, seen in Fig. 7, are linked to ${\text{C}}_{1}, {\text{C}}_{2}$ and ${\text{V}}_{\text{in}}$ in a series arrangement, while ${\text{S}}_{1}, {\text{S}}_{3}, {\text{S}}_{6}, {\text{S}}_{7}, {\text{S}}_{8}$ and ${\text{S}}_{12}$ are in conduction simultaneously. As a result, the output voltage ${\text{v}}_{\text{ab}}\hspace{0.17em}={\text{v}}_{\text{C}1}+{\text{v}}_{\text{C}2}+{\text{V}}_{\text{in}}= 2.5{\text{V}}_{\text{in}}$.

**Fig. 8:**
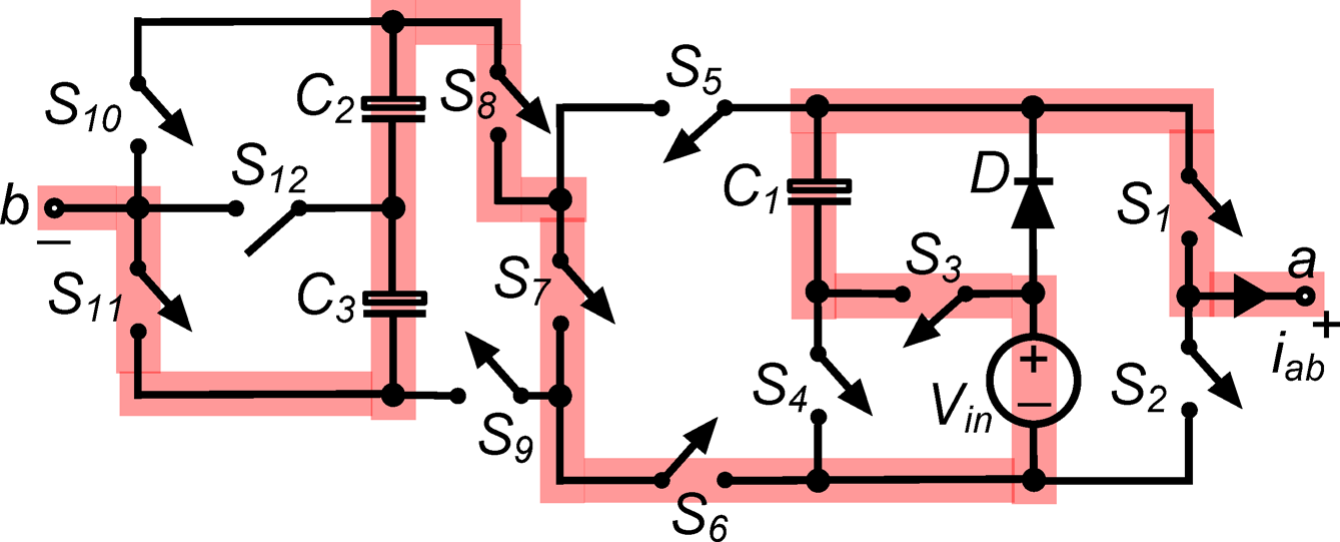
Working state ${\text{\O }}_{7}$ of the PI [${\text{v}}_{\text{ab}}=3{\text{V}}_{\text{in}}$].

*(vii) State* ${\O }_{7}$ : In the state illustrated in Fig. 8, ${\text{S}}_{1}, {\text{S}}_{3}, {\text{S}}_{6}, {\text{S}}_{7}, {\text{S}}_{8}$ and ${\text{S}}_{11}$ are all simultaneously conducting when the load is in series with ${\text{C}}_{1}, {\text{C}}_{2}, {\text{C}}_{3}$ and ${\text{V}}_{\text{in}}$. As a result, the output voltage ${\text{v}}_{\text{ab}}\hspace{0.17em}=\hspace{0.17em}{\text{v}}_{\text{C}1}+{\text{v}}_{\text{C}2}+{\text{v}}_{\text{C}3}+{\text{V}}_{\text{in}}= 3{\text{V}}_{\text{in}}$.

**Fig. 9:**
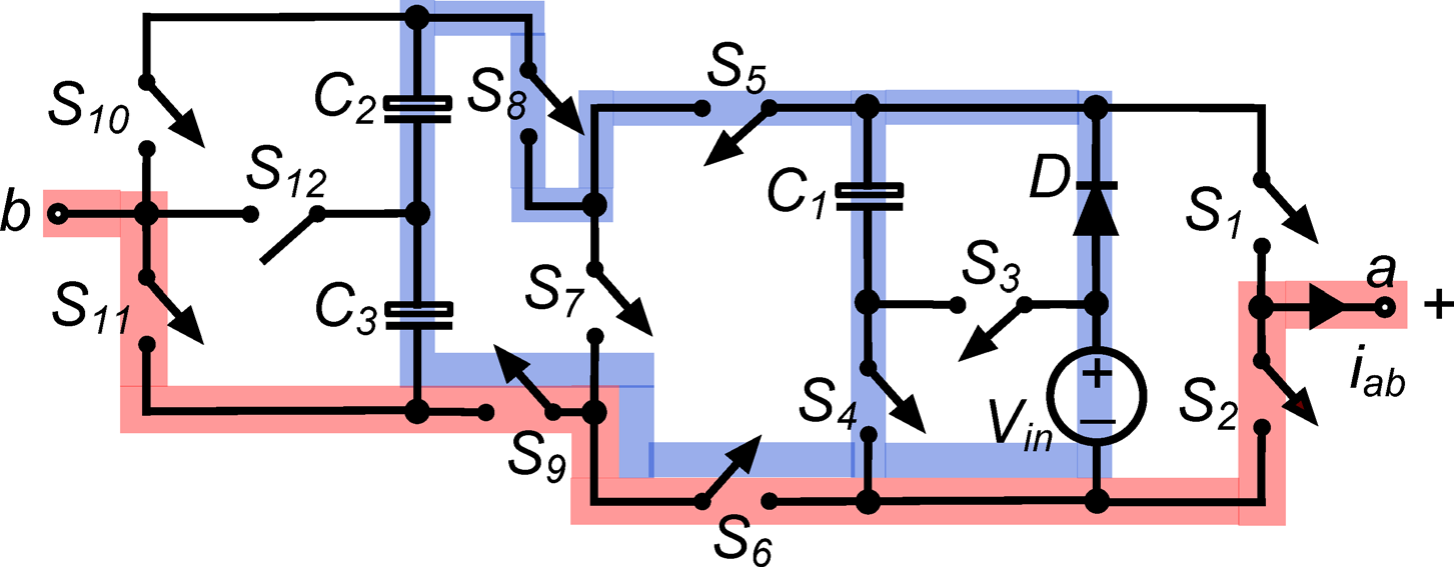
Working state ${\text{\O }}_{8}$ of the PI [${\text{v}}_{\text{ab}}=0$].

*(viii) State *${\O }_{8}$*:* In the state depicted in Fig. 9, switches of ${\text{S}}_{2}, {\text{S}}_{6}, {\text{S}}_{9}$ and ${\text{S}}_{11}$ all conduct simultaneously to provide the output voltage ${\text{v}}_{\text{ab}}\hspace{0.17em}=\hspace{0.17em}0$. Furthermore, ${\text{C}}_{1}$ is charged to ${\text{V}}_{\text{in}}$ by being placed in parallel with the dc source through diode $\text{D}$ with ${\text{S}}_{4}$ in conduction. Similar to this, the concurrent conduction of ${\text{S}}_{5}, {\text{S}}_{6}, {\text{S}}_{8}$ and ${\text{S}}_{9}$ connects the series combination of ${\text{C}}_{2}$ and ${\text{C}}_{3}$ in parallel with the dc source, charging each capacitor to $0.5{\text{V}}_{\text{in}}$.

**Fig. 10:**
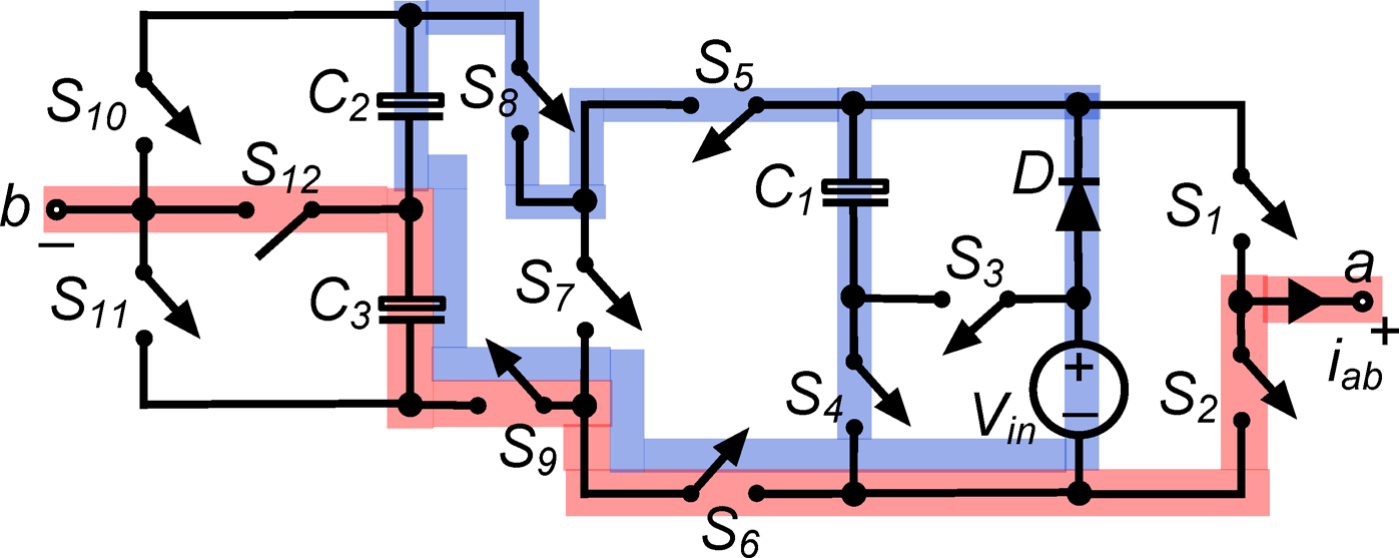
Working state ${\text{\O }}_{9}$ of the PI [${\text{v}}_{\text{ab}}=-0.5{\text{V}}_{\text{in}}$].

*(ix) State *${\O }_{9}$*:* The load is connected to ${\text{C}}_{3}$ in this state (Fig. 10), where ${\text{S}}_{2}, {\text{S}}_{6}, {\text{S}}_{9}\text{ and }{\text{S}}_{12}$ are all conducts simultaneously. ${\text{v}}_{\text{ab}}\hspace{0.17em}=\hspace{0.17em}-{\text{v}}_{\text{C}3}=-0.5{\text{V}}_{\text{in}}$ is the output voltage as a result. Additionally, ${\text{C}}_{1}$ is charged to ${\text{V}}_{\text{in}}$ through diode $\text{D}$, which is connected in parallel with the dc source with ${\text{S}}_{4}$ in conduction. Also, $0.5{\text{V}}_{\text{in}}$ is achieved by the capacitors ${\text{C}}_{2}$ and ${\text{C}}_{3}$, which are parallel with the input and conduct the switches ${\text{S}}_{5}, {\text{S}}_{6}, {\text{S}}_{8}\text{ and }{\text{S}}_{9}$ simultaneously.

**Fig. 11:**
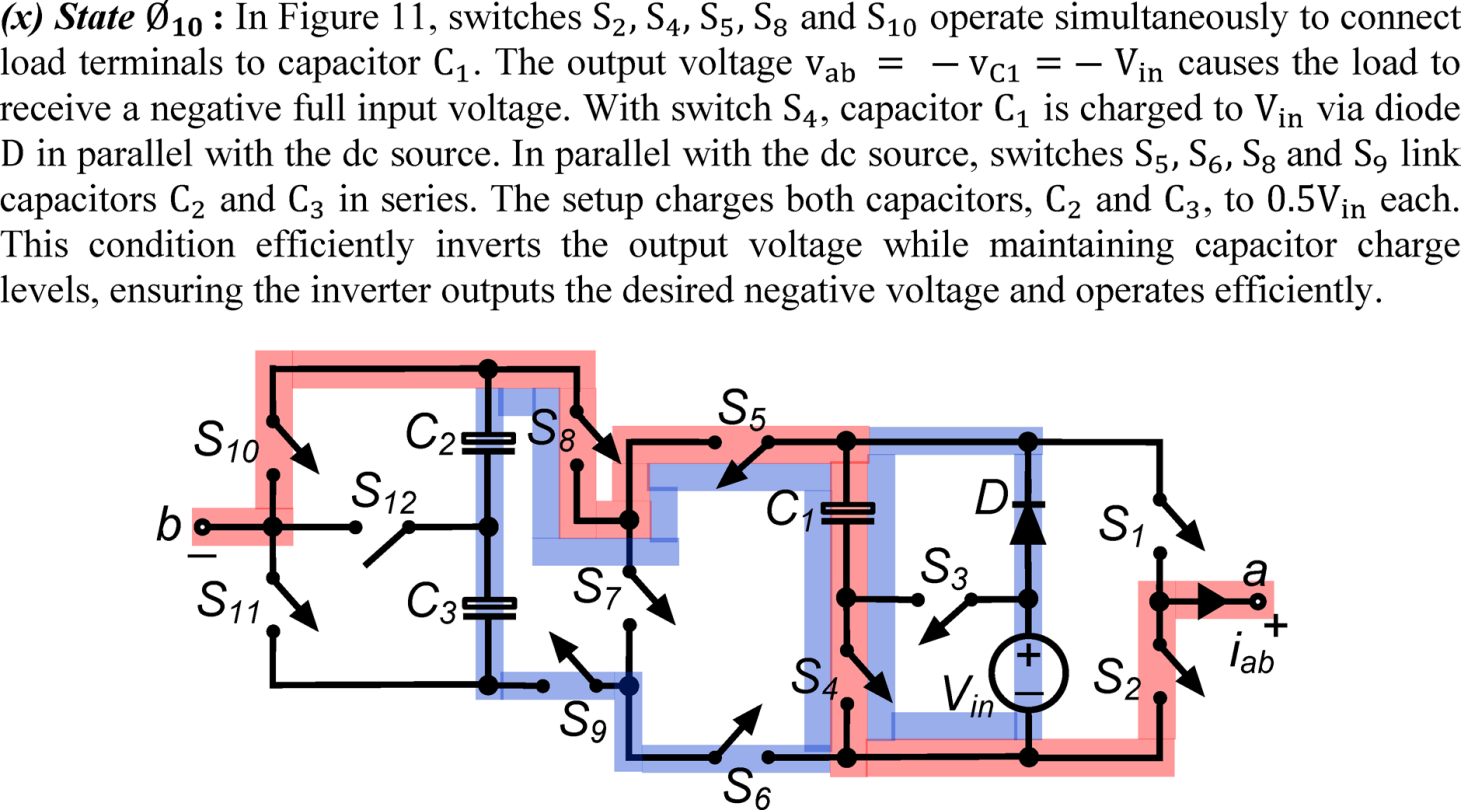
Working state ${\text{\O }}_{10}$ of the PI [${\text{v}}_{\text{ab}} = -{\text{V}}_{\text{in}}$].

*(x) State* ${\O }_{10}$ : In Fig. 11, switches ${\text{S}}_{2}, {\text{S}}_{4}, {\text{S}}_{5}, {\text{S}}_{8}$ and ${\text{S}}_{10}$ operate simultaneously to connect load terminals to capacitor ${\text{C}}_{1}$. The output voltage ${\text{v}}_{\text{ab}}\hspace{0.17em}=\hspace{0.17em}-{\text{v}}_{\text{C}1}=-{\text{V}}_{\text{in}}$ causes the load to receive a negative full input voltage. With switch ${\text{S}}_{4}$, capacitor ${\text{C}}_{1}$ is charged to ${\text{V}}_{\text{in}}$ via diode $\text{D}$ in parallel with the dc source. In parallel with the dc source, switches ${\text{S}}_{5}, {\text{S}}_{6}, {\text{S}}_{8}$ and ${\text{S}}_{9}$ link capacitors ${\text{C}}_{2}$ and ${\text{C}}_{3}$ in series. The setup charges both capacitors, ${\text{C}}_{2}$ and ${\text{C}}_{3}$, to $0.5{\text{V}}_{\text{in}}$ each. This condition efficiently inverts the output voltage while maintaining capacitor charge levels, ensuring the inverter outputs the desired negative voltage and operates efficiently.

**Fig. 12:**
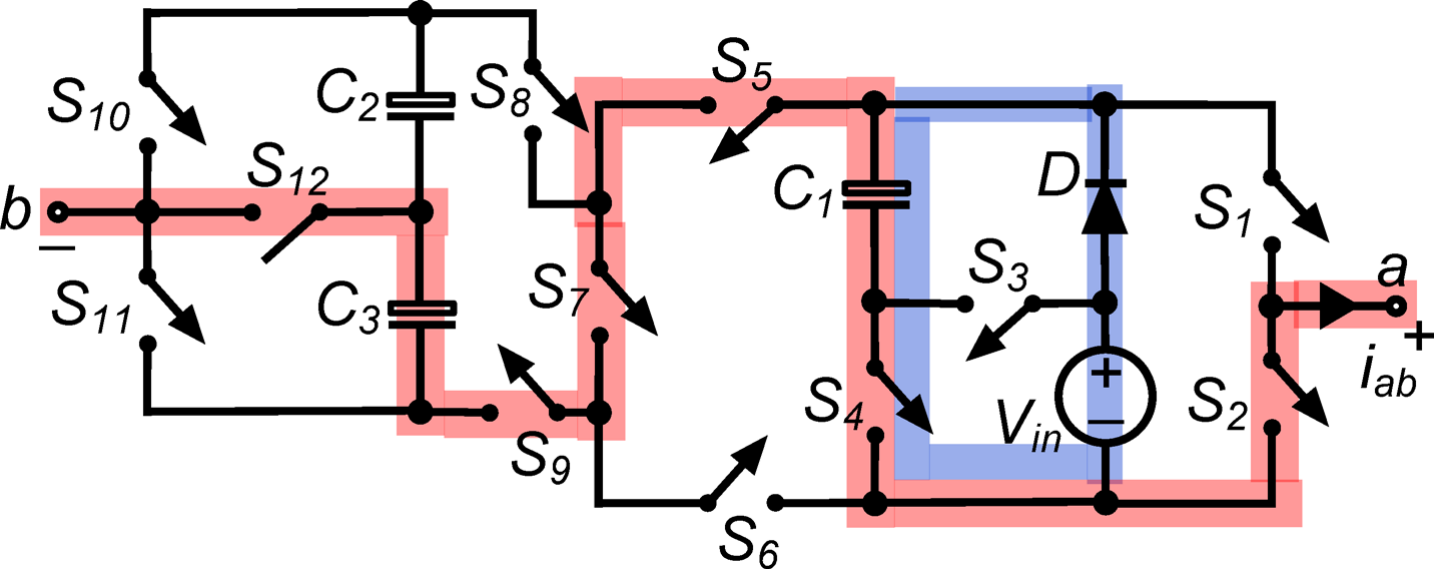
Working state ${\text{\O }}_{11}$ of the PI [${\text{v}}_{\text{ab}}=-1.5{\text{V}}_{\text{in}}$].

*(xi) State* ${\O }_{11}$ : In this state, as shown in Fig. 12, the load, ${\text{C}}_{1}$ and ${\text{C}}_{3}$ are in series, when simultaneous conduction of ${\text{S}}_{2}, {\text{S}}_{4}, {\text{S}}_{5}, {\text{S}}_{7}, {\text{S}}_{9}\text{ and }{\text{S}}_{12}$ occurs. Thus, output voltage ${\text{v}}_{\text{ab}}= -({\text{v}}_{\text{C}1}+ {\text{v}}_{\text{C}3}) = -1.5{\text{V}}_{\text{in}}$. Again, the conduction of ${\text{S}}_{4}$ connects ${\text{C}}_{1}$ in parallel with the DC source through diode ‘$\text{D}$’, and it is charged to ${\text{V}}_{\text{in}}$.

**Fig. 13:**
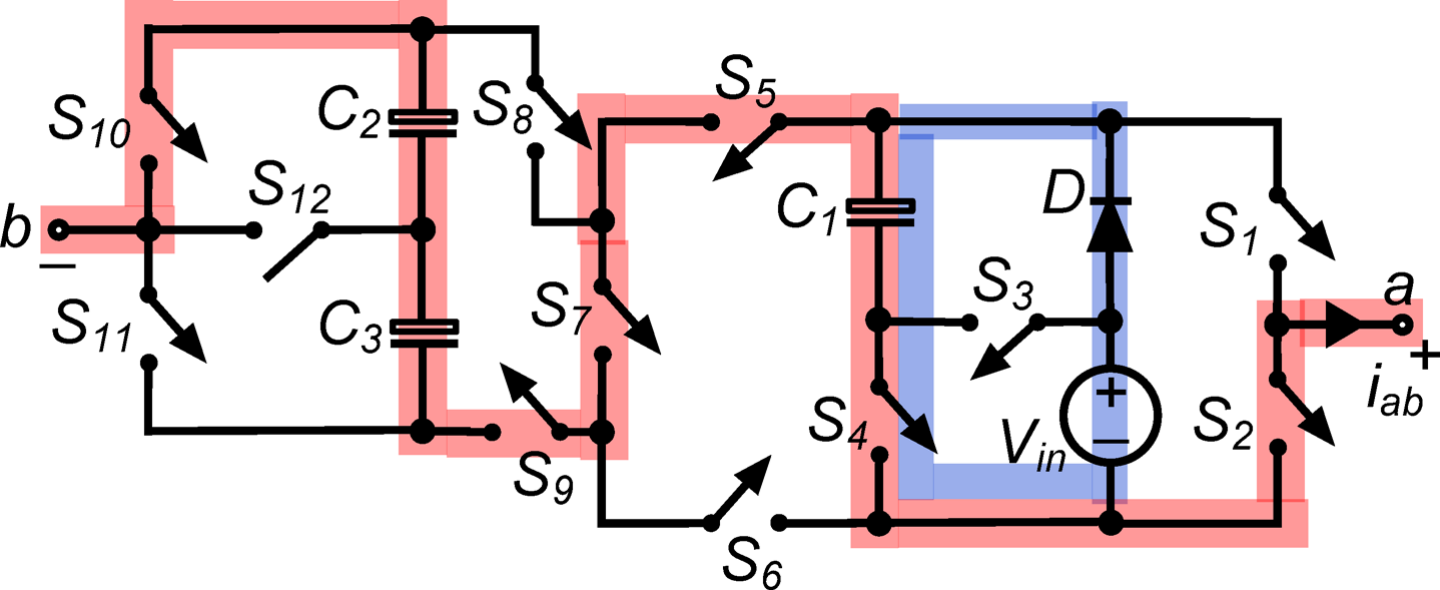
Working state ${\text{\O }}_{12}$ of the PI [${\text{v}}_{\text{ab}} = -2{\text{V}}_{\text{in}}$].

*(xii) State* ${\O }_{12}$ : As shown in Fig. 13, capacitors ${\text{C}}_{1} , {\text{C}}_{2} , {\text{C}}_{3}$ and load are in series through switches ${\text{S}}_{2}, {\text{S}}_{4}, {\text{S}}_{5}, {\text{S}}_{7}, {\text{S}}_{9}$ and ${\text{S}}_{10}$, resulting in an output voltage of ${\text{v}}_{\text{ab}}=-({\text{v}}_{\text{C}1}+ {\text{v}}_{\text{C}2}+ {\text{v}}_{\text{C}3})= -2{\text{V}}_{\text{in}}$. By conducting switch ${\text{S}}_{4}$, capacitor ${\text{C}}_{1}$ is connected in parallel with the dc source via diode $\text{D}$, allowing it to charge to ${\text{V}}_{\text{in}}$.

**Fig. 14:**
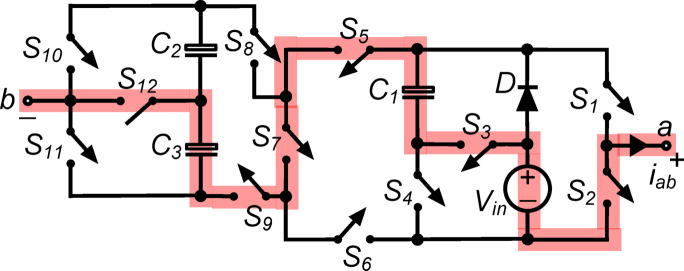
Working state ${\text{\O }}_{13}$ of the PI [${\text{v}}_{\text{ab}}=-2.5{\text{V}}_{\text{in}}$].

*(xiii) State* ${\O }_{13}$: As shown in Fig. 14, the load and capacitors ${\text{C}}_{1}$ and ${\text{C}}_{3}$ are connected consecutively, and the input voltage ${\text{V}}_{\text{in}}$ via switches ${\text{S}}_{2}, {\text{S}}_{3}, {\text{S}}_{5}, {\text{S}}_{7}, {\text{S}}_{9},$ and ${\text{S}}_{12}$. Output voltage ${\text{v}}_{\text{ab}}=-({\text{v}}_{\text{C}1}+ {\text{v}}_{\text{C}3}+ {\text{V}}_{\text{in}})=-2.5{\text{V}}_{\text{in}}$ is delivered to the load as a negative voltage 2.5 times the input voltage.

**Fig. 15:**
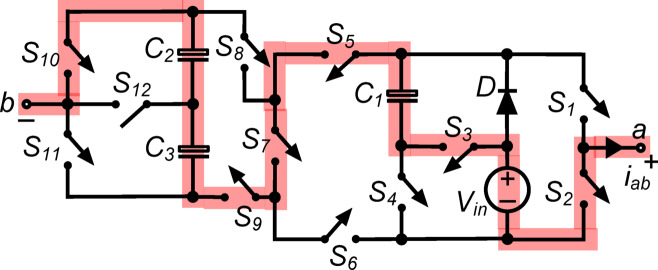
Working state ${\text{\O }}_{14}$ of the PI [${\text{v}}_{\text{ab}}=-3{\text{V}}_{\text{in}}$].

*(xiv) State* ${\O }_{14}$ : Fig. 15 shows the capacitors ${\text{C}}_{1} , {\text{C}}_{2}$, and ${\text{C}}_{3}$ are connected in series with the load and the input voltage ${\text{V}}_{\text{in}}$ via switches ${\text{S}}_{2}, {\text{S}}_{3}, {\text{S}}_{5}, {\text{S}}_{7}, {\text{S}}_{9},$ and ${\text{S}}_{10}$. In this state, the output voltage ${\text{v}}_{\text{ab}}=-({\text{v}}_{\text{C}1}+{\text{v}}_{\text{C}2}+{\text{v}}_{\text{C}3}+{\text{V}}_{\text{in}})=-3{\text{V}}_{\text{in}}$ giving a negative voltage to the load three times the input voltage.

Thus, the AC terminals may obtain 13 voltage levels for both directions of load current regardless of load type—resistive, inductive, or capacitive. This verifies the proposed structure works under diverse loads. It is observed that capacitor ${C}_{1}$ is connected in parallel with the input DC source in ten of the fourteen states; however, in six of the states, the combination of capacitors ${C}_{2}$ and ${C}_{3}$ is in parallel with the input DC source, preserving its charge. This property keeps three capacitors self-balance. Since each capacitor is in a non-charging phase, the amount each capacitor discharges is dependent on the time spent in each state, the c3urrent across the load, and the angle between the voltage and current. This characteristic is given as follows.

1 $${\text{Q}}_{{\text{C}}} = \int\limits_{{{\text{t}}_{{\text{x}}} }}^{{{\text{t}}_{{\text{y}}} }} {{\text{I}}_{{{\text{ab}}}} \sin \left( {2\uppi {\text{f}}_{{\text{o}}} {\text{t}} - \upvarphi } \right){\text{dt}}} { }$$

In this analysis, $f_{o}$ represents power frequency, φ represents load power factor angle, $I_{ab}$ is the peak load current ${i}_{ab}$, and (t_y_ − t_x_) shows the duration of the operating situation. In order to ensure that the capacitors remain self-balanced without the need for additional control, the value of capacitors is ascertained and determined as per the load requirement of a particular application. To minimize voltage ripples and deliver the specified load power, the capacitance needs to be large enough. The required capacitance can be calculated as follows:

2 $$\begin{array}{*{20}c} { C = \frac{{\text{P}}}{{2{\pi *}2{\text{f}}_{{\text{o}}} {*}\left( {\Delta {\text{V}}_{{\text{c}}} } \right){\text{*V}}_{{{\text{ab}}}}^{2} }}} \\ \end{array}$$

where ${\text{P}}$ is active power, V_ab_ is the root-mean-squared value of the load voltage v_ab_ and ΔV_C_ is the capacitor voltage ripple.

Thus, the designed SCMLI, has the following valid conditions:

3 $$\begin{array}{*{20}c} {{\text{No}}{\text{. of levels}}, {\text{N}}_{{\text{L}}} = 13 } \\ \end{array}$$

4 $$\begin{array}{*{20}c} {{\text{No}}{\text{. of main power switches}}, {\text{N}}_{{\text{S}}} = 13 } \\ \end{array}$$

5 $$\begin{array}{*{20}c} {{\text{No}}{\text{. of main diodes}}, {\text{N}}_{{\text{D}}} = 13} \\ \end{array}$$

6 $$\begin{array}{*{20}c} {{\text{No}}{\text{. of auxiliary diodes}}, {\text{N}}_{{{\text{AD}}}} = 1} \\ \end{array}$$

7 $$\begin{array}{*{20}c} {{\text{No}}{\text{. of gate driver units}}, {\text{N}}_{{{\text{GD}}}} = 12 } \\ \end{array}$$

8 $$\begin{array}{*{20}c} {{\text{No}}{\text{. of input dc sources}}, {\text{N}}_{{{\text{IS}}}} = 1 } \\ \end{array}$$

9 $$\begin{array}{*{20}c} {{\text{No}}{\text{. of capacitors}}, {\text{N}}_{{\text{C}}} = 3} \\ \end{array}$$

10 $$\begin{array}{*{20}c} {{\text{Voltage gain}}, {\text{V}}_{{\text{G}}} = 3 } \\ \end{array}$$

Here, the bidirectional-blocking-bidirectional-conducting switch is accounted for using two power switches in a common emitter connection. Hence, the total number of power switches in (4) is taken as thirteen. Also, as a common emitter connection is used, a common gate driver unit will suffice, and hence, the total number of gate driver units is taken to be twelve in (7) above.

## Comparison with other scmlis

While classical topologies typically achieve a voltage gain of unity, switched capacitor-based structures provide gains exceeding unity. Therefore, the PI is evaluated against recent literature of various switched capacitor-based structures. Table 2 outlines a comparison with contemporary topologies based on device count, while Table 3 extends this comparison to TSV and PIV requirements, both crucial for reliability and applicability^2,11^. Regardless of the total number of output levels produced, a standardized matching between structures can be obtained through the evaluation of the number of components employed per synthesized output level. Analysis presented in the ninth column of Table 2 shows the component efficiency of each structure, which allows for an unbiased assessment of their relative performance.

**Table 2:** Comparison of the PI with other switched capacitors-based topologies.

Literature	${\text{N}}_{\text{L}}$	${\text{N}}_{\text{IS}}$	${\text{N}}_{\text{S}}$	${\text{N}}_{\text{D}}$	${\text{N}}_{\text{AD}}$	${\text{N}}_{\text{GD}}$	${\text{N}}_{\text{C}}$	Device count per level
^4^	13	2	16	16	2	16	4	4.30
^5^	13	1	10	10	4	10	4	3.00
^6^	5	1	9	9	0	9	1	5.80
^7^	9	1	10	10	1	8	2	3.55
^8^	9	1	12	12	0	11	2	4.22
^9^	7	1	10	10	0	10	2	4.71
^10^	5	1	6	6	2	6	1	4.40
^11^	7	1	16	16	0	14	2	7.00
^12^	5	1	6	6	2	6	2	4.60
^13^	5	1	9	9	1	8	1	5.80
^14^	5	1	6	6	1	6	1	4.20
^15^	5	1	12	12	0	12	2	7.80
^16^	5	1	7	7	3	7	2	5.40
^17^	9	1	11	11	0	10	2	3.88
^8^	9	1	12	12	0	11	2	4.22
^18^	9	1	11	11	0	10	2	3.89
^19^	13	1	14	14	1	14	3	3.62
^20^	13	1	13	13	2	13	3	3.46
^21^	9	1	11	11	0	10	2	3.89
^22^	13	1	13	13	2	13	3	3.46
^23^	13	1	13	13	2	13	3	3.46
^24^	13	1	15	15	-	15	3	3.77
^25^	5	1	8	8	1	8	2	5.60
^26^	13	2	11	11	1	11	2	2.92
Proposed	13	1	13	13	1	12	3	3.30

**Table 3:** Evaluation of the suggested topology against other SCMLI topologies based on TSV, PIV for input DC voltage ${V}_{in}$, and cost function.

Reference	${\text{N}}_{\text{L}}$	TSV	PIV	$\text{X}$	$\text{Y}$	C.F
^4^	13	34V_in_	6V_in_	2.615	0.462	13.538
^5^	13	33V_in_	5V_in_	2.538	0.385	5.462
^6^	5	9V_in_	V_in_	1.800	0.200	7.400
^7^	9	11V_in_	2V_in_	1.222	0.222	4.667
^8^	9	11V_in_	V_in_	1.222	0.111	5.333
^9^	7	18V_in_	3V_in_	2.571	0.428	7.143
^10^	5	12V_in_	2V_in_	2.400	0.400	6.600
^11^	7	16V_in_	2V_in_	2.285	0.285	9.143
^12^	5	8V_in_	V_in_	1.600	0.200	6.000
^13^	5	9V_in_	V_in_	1.800	0.200	7.400
^14^	5	11V_in_	2V_in_	2.200	0.400	6.200
^15^	5	20V_in_	V_in_	4.000	0.200	11.600
^16^	5	9V_in_	V_in_	1.800	0.200	7.000
^17^	9	10V_in_	V_in_	1.111	0.111	4.889
^8^	9	11V_in_	V_in_	1.222	0.111	5.333
^18^	9	10V_in_	V_in_	1.111	0.111	4.889
^19^	13	28V_in_	3V_in_	2.154	0.231	5.692
^20^	13	33V_in_	3V_in_	2.538	0.231	5.923
^21^	9	10V_in_	V_in_	1.111	0.111	4.889
^22^	13	32V_in_	V_in_	2.462	0.077	5.846
^23^	13	33V_in_	3V_in_	2.538	0.231	5.923
^24^	13	30V_in_	3V_in_	2.308	0.231	6.000
^25^	5	12V_in_	2V_in_	2.400	0.400	7.800
^26^	13	31V_in_	5V_in_	2.385	0.385	5.154
Proposed	13	17V_in_	2V_in_	1.307	0.153	4.538

From Table 2, it is evident that the PI entails a reduced number of components per level in contrast to^5,26^, with the latter exhibiting even lower component counts. However, on examining Table 3, the TSV and PIV of topologies in^5,26^ are high as compared to the PI. In fact, a methodology to incorporate the component count and TSV in a single parameter (called cost function ‘CF’) is presented in^7^ for evaluating a topology and is widely used to determine the merit of an SCMLI^5^. It is defined as^7^:

11 $$\begin{array}{*{20}c} {{\text{CF}} = \frac{{{\text{N}}_{{{\text{IS}}}} }}{{{\text{N}}_{{\text{L}}} }}*\left( {{\text{N}}_{{\text{S}}} + {\text{N}}_{{\text{D}}} + {\text{N}}_{{{\text{AD}}}} + {\text{N}}_{{{\text{GD}}}} + {\text{N}}_{{\text{C}}} + {\text{TSV}}} \right) } \\ \end{array}$$

**Fig. 16:**
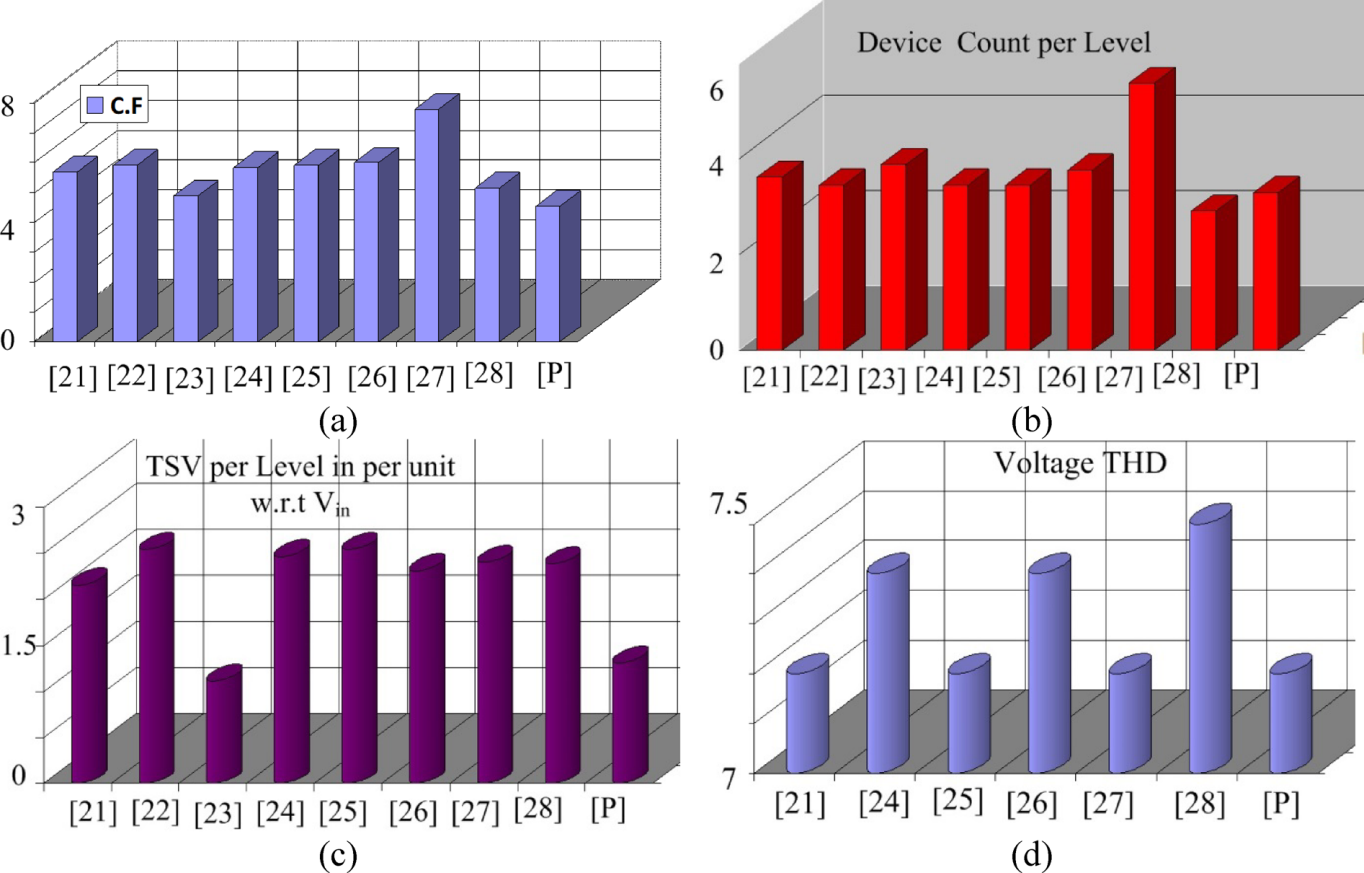
Graphical representation (**a**) CF (**b**) Device count per level (**c**) TSV level per unit w.r.t ${V}_{in}$ (**d**) Voltage THD.

Table 3 records the C.F. for all the topologies under comparison, and it can be observed that the suggested topology is the most competent amongst the contemporary ones in terms of C.F. The THD comparison with similar structures is presented in Table 4, highlighting the superior performance of the proposed design. Figure 16 illustrates the graphical representation of CF, device count per level, TSV per level per unit with respect to ${V}_{in}$ (X), PIV per level per unit with respect to ${V}_{in}$(Y) and voltage THD.

**Table 4:** THD comparison with similar structure.

Reference	Voltage THD	Current THD
^19^	7.2	0.7
^22^	7.4	0.8
^23^	7.2	0.6
^24^	7.4	0.8
^25^	7.2	0.6
^26^	7.5	1
[P]	7.2	0.6

## Switching methodology

**Fig. 17:**
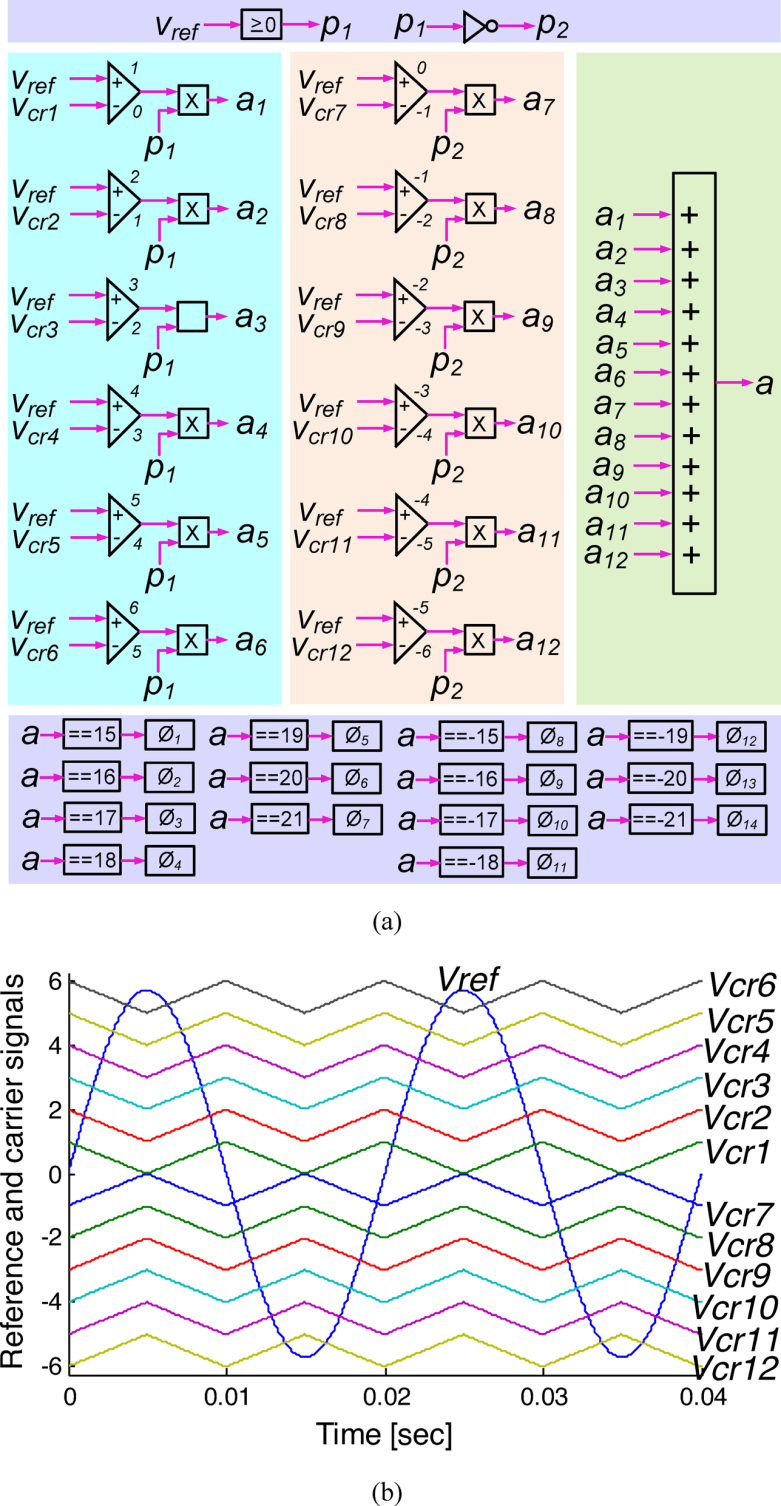

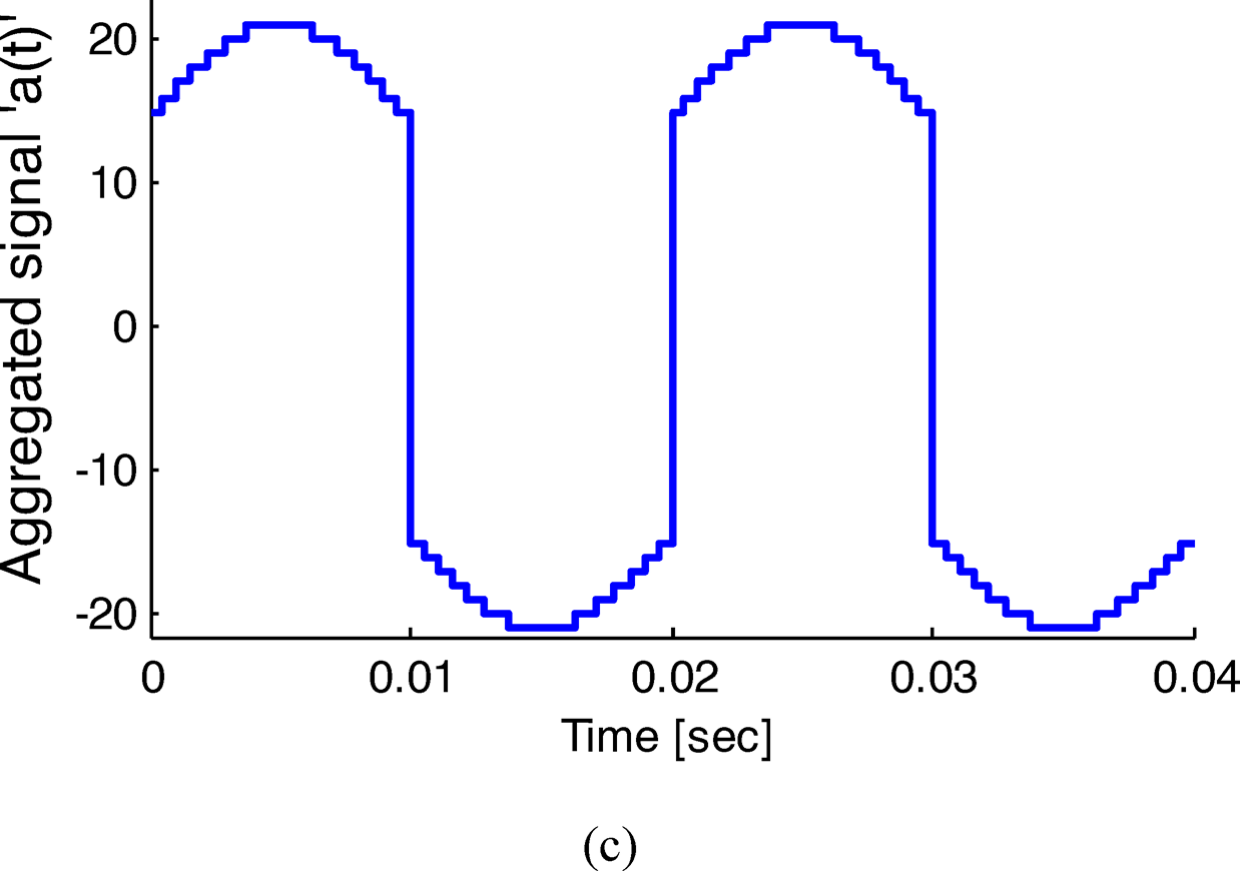
(**a**) Switching strategy of designed SCMLI; (**b**) ${\text{V}}_{\text{ref}}$ and ${\text{V}}_{\text{crj}}$; and (**c**) Aggregated signal ‘a’.

The suggested topology can be generalized for different multilevel inverter topologies with proper modifications. Thus, this work uses the multicarrier PWM scheme proposed in^30^ because it has the possibility of using both null states. The technique, as depicted in Fig. 17(a), employed 12-triangular high-frequency waveforms ${\text{V}}_{\text{crj}} \left\{\text{j }= 1\text{ to }12\right\}$ as carriers in phase opposition. The waveforms are exhibited at low frequency for clarity. A sinusoidal waveform ${\text{V}}_{\text{ref}}$ with frequency ${\text{f}}_{\text{o}}$ serves as the reference signal. In Fig. 17(b), reference and carrier signals are shown. Comparators continuously compare reference and carrier signals. Comparators output values of 1, 2, 3, 4, 5, 6, 0, − 1, − 2, − 3, − 4, and -5 when ${\text{V}}_{\text{ref}}$ $>{\text{V}}_{\text{crj}}$. On the other hand, comparators produce the corresponding outputs as 0, 1, 2, 3, 4, 5, − 1, − 2, − 3, − 4, − 5, and − 6 if the ${\text{V}}_{\text{ref}} <{\text{V}}_{\text{crj}}$. An aggregated signal ‘a’ is obtained by adding signals ${\text{a}}_{\text{j}}$ $\{\text{j }= 1\text{ to }12\}$, as shown in Fig. 17(c).

The signal ‘a’ is an aggregated waveform consisting of seven discrete positive levels, ranging from 15 to 21 in increments of + 1, and seven discrete negative levels, ranging from –15 to –21 in decrements of –1. The triggering pulses are produced in a 1:1 relationship with the levels at the output waveform as depicted in Fig. 17(a).

## Power losses

Three forms of power losses occur in a switched-capacitor-based MLI: capacitor, switching, and conduction^9,10,31^. The following losses are briefly discussed:

*(a) Power losses in capacitors*: The following equation^9^ calculates capacitor voltage ripple for each inverter operational state:

12 $$\begin{array}{*{20}c} {\Delta {\text{V}}_{{\text{C}}} = \frac{1}{{\text{C}}}\mathop \smallint \limits_{{{\text{t}}_{{\text{x}}} }}^{{{\text{t}}_{{\text{y}}} }} {\text{i}}_{{\text{C}}} {\text{dt}}} \\ \end{array}$$

Here, ${\text{i}}_{\text{C}}$ is the current through a switched capacitor, (t_y_—t_x_) is the discharging time span, and C is the capacitance value. Hence, for each of the states used for level generation, the power losses due to capacitor ripples as (${f}_{o}$ being the power frequency):

13 $$\begin{array}{*{20}c} {{\text{P}}_{{\text{Ripple Losses}}} = \frac{{{\text{f}}_{{\text{o}}} }}{2}C\left( {\Delta {\text{V}}_{{\text{c}}} } \right)^{2} } \\ \end{array}$$

In switched capacitors of the topology, power losses also take place due to equivalent series resistance (ESR) of a given capacitor (${R}_{ESR}$). Thus, the conduction losses in each switched capacitor can be calculated as:

14 $$\begin{array}{*{20}c} {{\text{P}}_{{\text{Conduction losses in C}}} = \frac{{{\text{R}}_{{{\text{ESR}}}} {\text{f}}_{{\text{o}}} }}{2}\mathop \smallint \limits_{{{\text{t}}_{{\text{x}}} }}^{{{\text{t}}_{{\text{y}}} }} {\text{i}}^{2}_{{\text{C}}} {\text{dt}}} \\ \end{array}$$

So, adding (13) and (14) gives the total power losses taking place in a given switched capacitor of an SCMLI.

*(b) Switching power losses:* During switching in a power semiconductor device, the intrinsic delays lead to switching losses. For each of the power switches, the losses during turning ON and OFF can be obtained as ^9^:

15 $$\begin{array}{*{20}c} {{\text{P}}_{{\text{Switching Losses}}} = \frac{1}{6} {\text{V}}_{{{\text{in}}}} i\left( {\text{t}} \right)\left\{ {{\text{t}}_{{{\text{ON}}}} + {\text{t}}_{{{\text{OFF}}}} } \right\}{\text{f}}_{{\text{s}}} } \\ \end{array}$$

where, V_in_ = voltage stress bear by the switch during its OFF state; i(t) = current bearing capability of the switch during conduction; ${\text{t}}_{\text{ON}}$= the time at which the switch is ON; ${\text{t}}_{\text{OFF}}$ = the time at which the switch is OFF; ${f}_{s}$= switching frequency.

*(c) Conduction sosses*: Conduction losses for the transistor and diode part of a given power switch are obtained using the following equations with the description of the variables given in^17^:

16 $$\begin{array}{*{20}c} {{\text{P}}_{{\text{Conduction Losses of Transistor}}} = {\text{V}}_{{{\text{on}},{\text{ sw}}}} {\text{I}}_{{{\text{sw}},{\text{ avg}}}} + {\text{R}}_{{{\text{on}},{\text{ sw}}}} {\text{I}}_{{{\text{sw}},{\text{ rms}}}}^{2} } \\ \end{array}$$

17 $$\begin{array}{*{20}c} {{\text{P}}_{{\text{Conduction Losses of Diode}}} = {\text{V}}_{{{\text{on}},{\text{ d}}}} {\text{I}}_{{{\text{d}},{\text{ avg}}}} + {\text{R}}_{{{\text{on}},{\text{d}}}} {\text{I}}_{{{\text{d}},{\text{rms}}}}^{2} } \\ \end{array}$$

Therefore, the power losses of the proposed SCMLI can be calculated as an aggregation of these losses. The losses occurred by individual switches are analyzed and presented in Table 5.

**Table 5:** Individual losses (in Watt).

Losses	*S*_*1*_	*S*_*2*_	*S*_*3*_	*S*_*4*_	*S*_*5*_	*S*_*6*_	*S*_*7*_	*S*_*8*_	*S*_*9*_	*S*_*10*_	*S*_*10*_	*S*_*12*_
Switching loss	0.273	0.221	1.33	0.622	0.78	0.73	1.01	1.02	0.597	0.624	0.385	0.534
Conduction loss	0.87	0.906	1.99	2.44	1.27	1.30	1.54	1.054	4.09	3.99	4.20	0.87

## Experimental verification

**Fig. 18:**
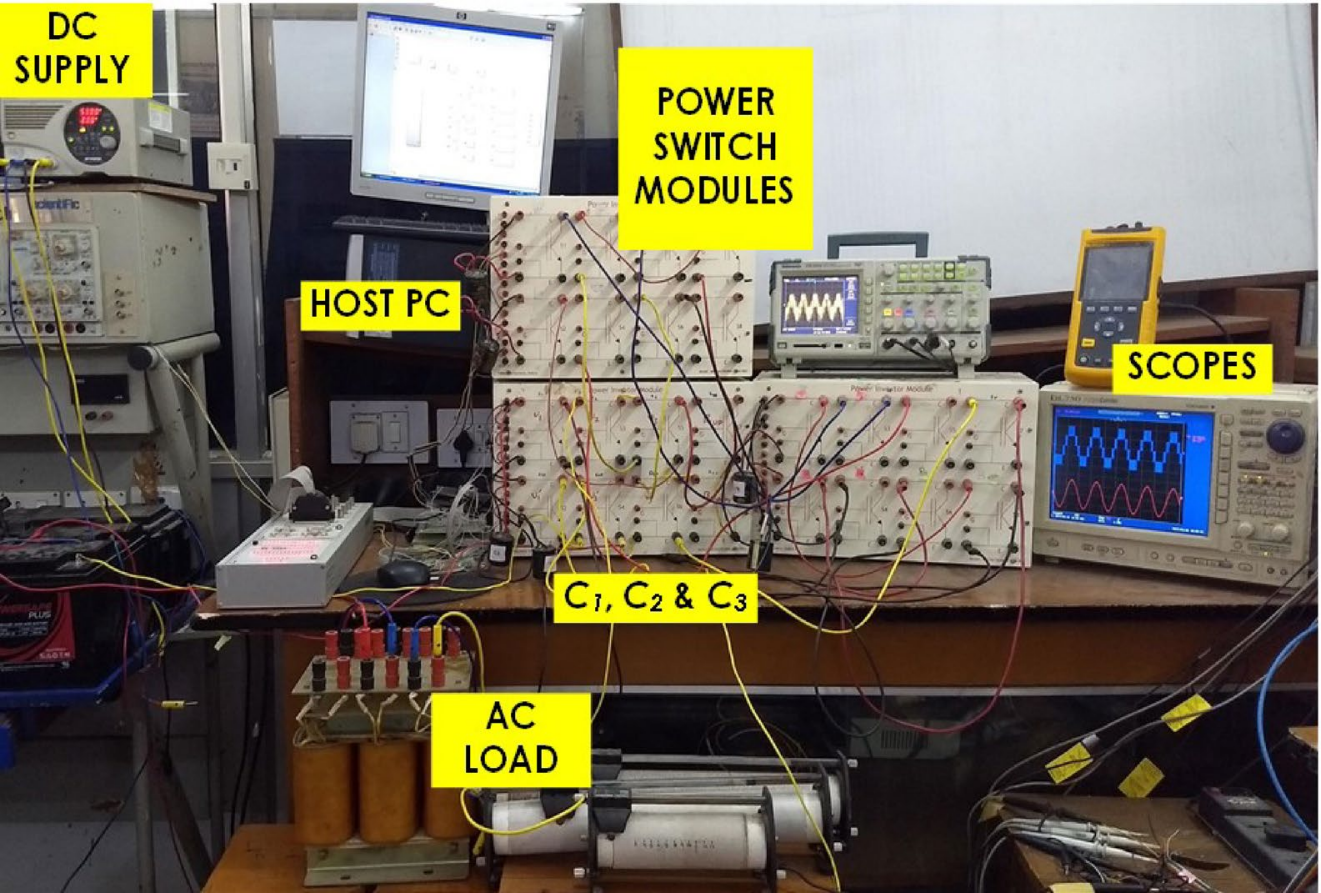
Prototype of the PI.

To experimentally verify the designed converter, a lab test bench was implemented with discrete switches, such as IRF460 MOSFETs, and appropriate gate drivers, as depicted in Fig. 18. The input voltage was calibrated to $100\text{ V}$, and capacitances of $2200$, have been adopted for ${\text{C}}_{1}$, ${\text{C}}_{2}$ and ${\text{C}}_{3}$. The dSPACE DS1103 was employed to create a gate pulse. The carrier and reference signal were at 1 kHz and 50 Hz, respectively, with a modulation index of 0.95. The load terminals were connected to an inductive load, a 50-Ω resistor ($\text{R}$), and a 170 mH inductor ($\text{L}$).

**Fig. 19:**
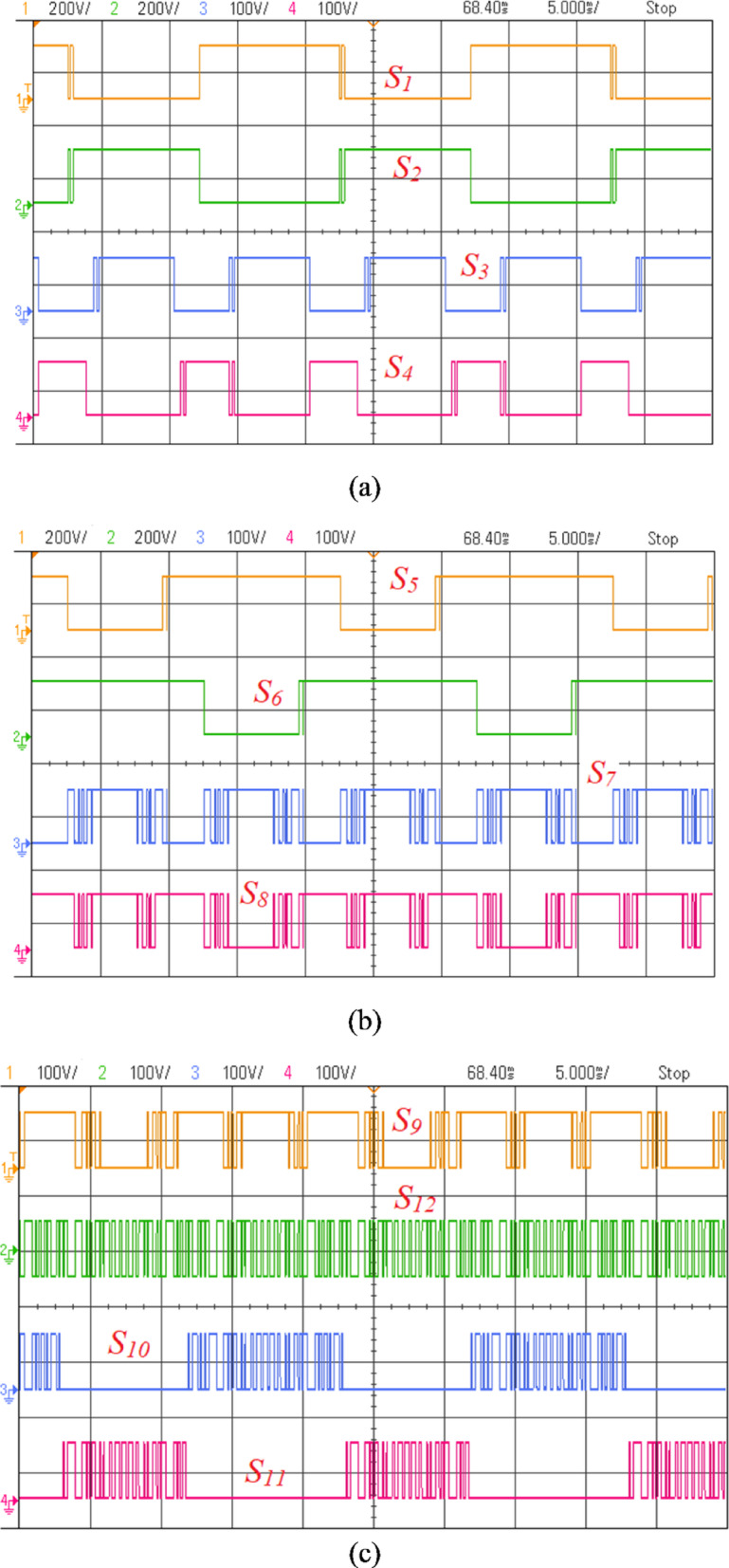
(**a**–**c**) Voltage waveforms across power switches.

The voltage waveforms across the power switches are illustrated in Fig. 19, demonstrating adherence to the PIV values specified in Table 1, with ${\text{V}}_{\text{in}}$ set at 100 V.

**Fig. 20:**
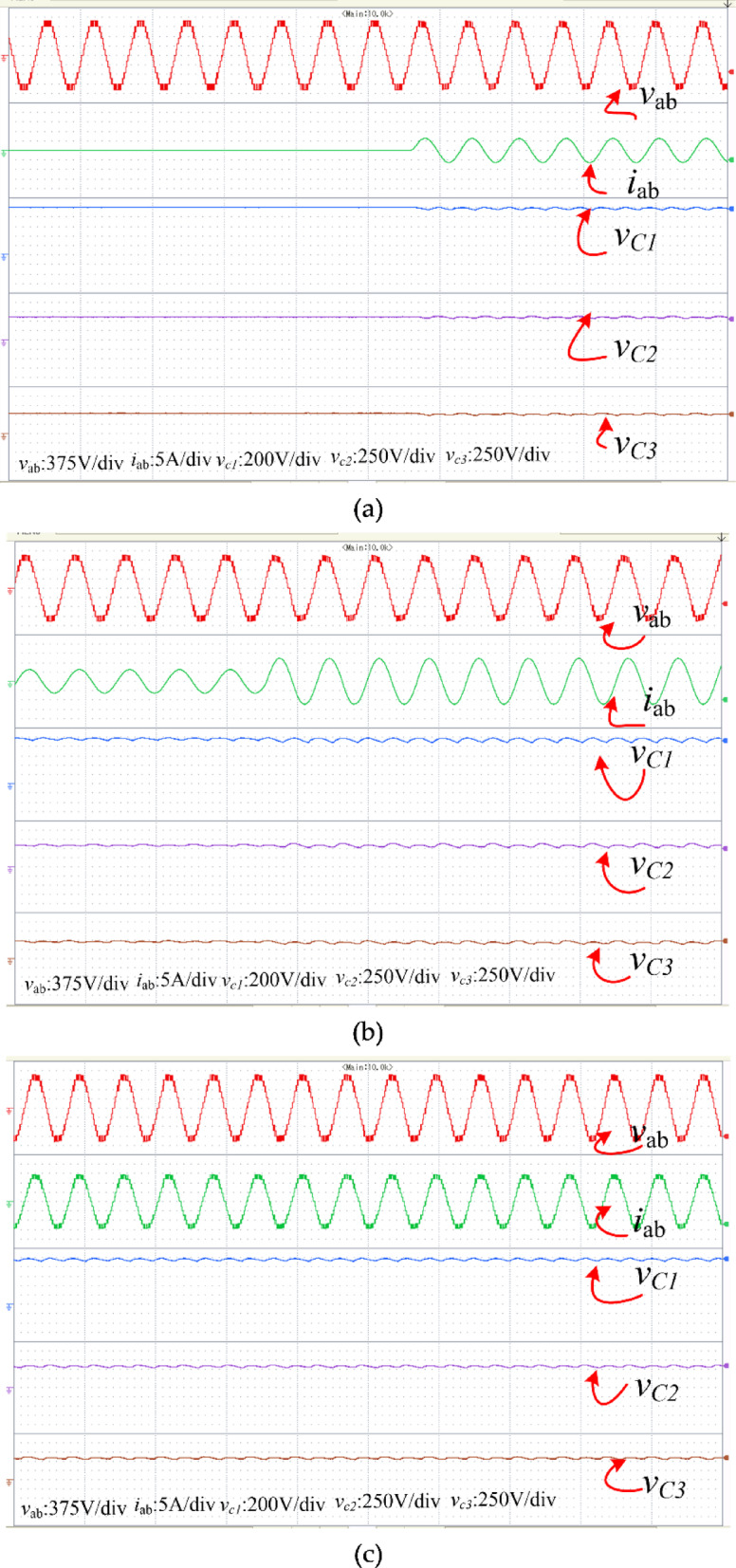

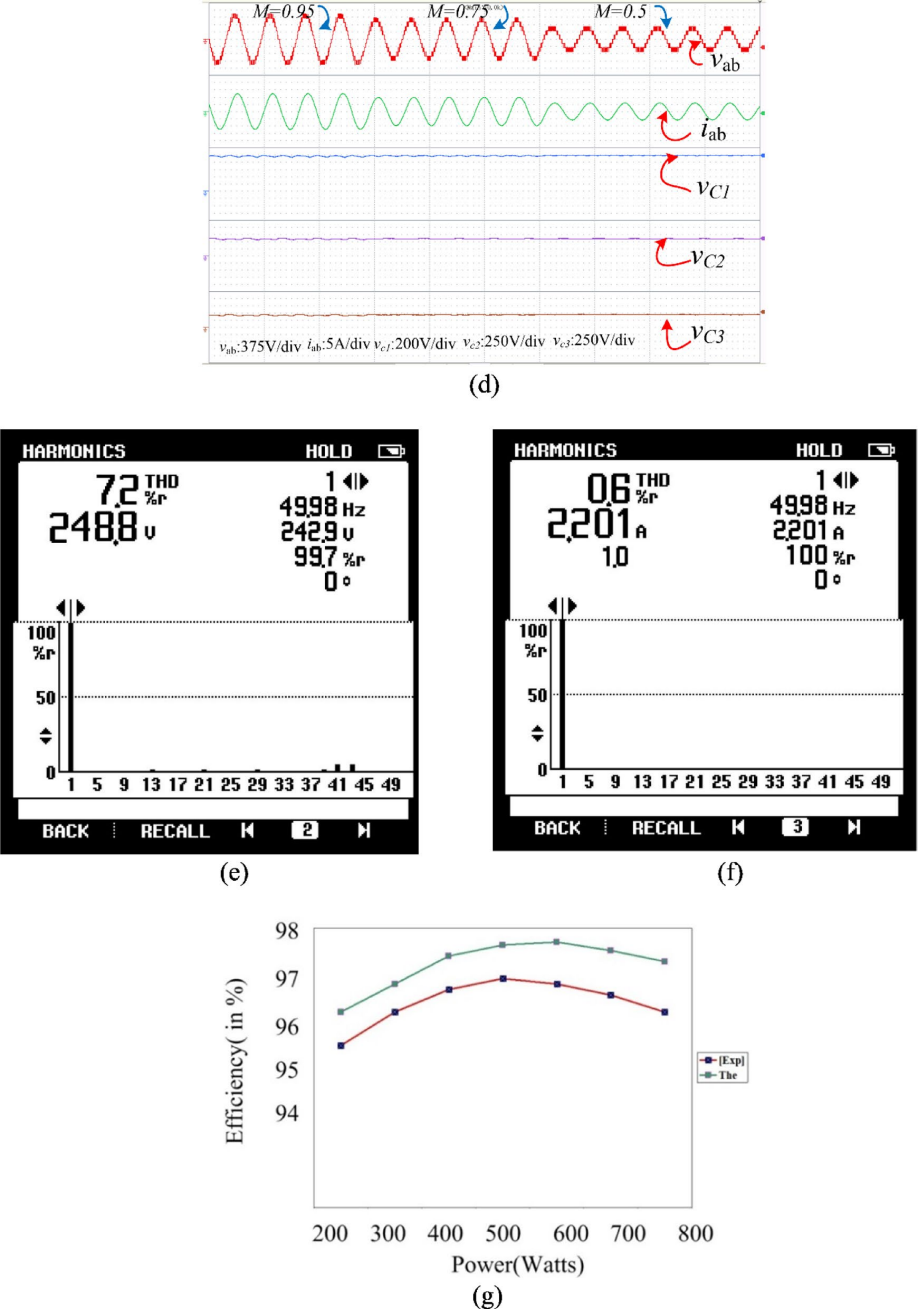
Experimental results: (**a**) Start-in response with waveforms of load voltage, load current, and capacitor voltages; and (**b**) Waveforms when the load is increased. (**c**) R-load (**d**) Change in Modulation index (**e**) THD of the output voltage and (**f**) THD of Load current (**g**) Efficiency comparison.

Figure 20(a) depicts the start-up response of the inverter, where the capacitors C₁, C₂, and C₃ achieve self-balancing at 100 V, 50 V, and 50 V, respectively. As shown in Fig. 20(b), even when the load varies, the voltage waveforms remain unchanged. The output is a 13-level waveform with 50 V step increments, as anticipated. Hence, the feasibility of the PI is confirmed through its performance and the capacitors’ ability to self-balance. Figure 20(c) presents the results for a purely resistive load. It is observed that under resistive loading conditions, the capacitors maintain self-balancing, and the output voltage remains stable. Figure 20(d) illustrates the results under varying modulation index conditions. It is observed that despite the change in modulation index, the capacitors sustain self-balancing, while the output voltage adjusts correspondingly. The harmonic spectra of the voltage and current waveforms are illustrated in Fig. 20(e) and (f). The corresponding THD values for the voltage and current waveforms are 7.2% and 0.6%, respectively. Figure 20(g) presents both theoretical and experimental efficiencies, indicating that the experimental efficiency, at 96.7%, is slightly lower than theoretical value.

SBased on the literature review, MLIs have potential applications in the following areas:

(i) The power distribution system for high-frequency alternating current (HFAC): The elimination of filter stages and rectifiers from high-frequency alternating current (HFAC) power distribution systems (PDS) enhances power density, heat distribution, efficiency, and reduces the number of components compared to typical direct current (DCPDS) systems^26,30^. HFAC PDS improves system efficiency and finds use in electric vehicles, lights, microgrids, computers, and telecommunications^31^. One common method for making HFAC PDS more reliable is to use SCMLIs^4^.

(ii) Based on photovoltaic power generation systems: Photovoltaic systems produce minimal electricity, necessitating voltage amplification for grid integration. This can be accomplished using PV module cascading, DC-DC converters, or step-up transformers on the inverter’s AC side. However, these strategies elevate expenses, mass, and inefficiencies. SCMLIs provide benefits such as capacitor self-balancing, decreased filtering, enhanced voltage gain, and grid-compatible waveforms^9,27^.

(iii) Electric vehicle traction system (EVTS): EVTS are generally arranged in two configurations: (a) a direct connection from the battery to the inverter or (b) a connection from the battery- DC-DC converter. The former necessitates more series cells for elevated DC-link voltage, whereas the latter escalates expense and complexity. SCMLIs effectively tackle these difficulties, improving output voltage and system speed^7,28^.

## Conclusion

This paper presents a single-phase thirteen-level inverter with three capacitors for three-fold voltage boosting capability. The proposed switched capacitor inverter presents PIVs much lower than the operating voltage for most of the power-switching devices. The suggested topology and control strategy enables the capacitors to self-balance their voltage. A comparison with the existing topologies shows that the proposed design provides a smaller number of components, a lesser total standing voltage, and a more economical cost. The findings demonstrate that the proposed 13-level SC-MLI not only meets the harmonic standards but also offers a reliable and efficient solution with fewer components, making it a competitive option for high-performance multilevel inverter applications. Due to its high-resolution AC waveforms with increased output voltage, this inverter is ideal for applications where low voltage power supply is used as DC source input, such as integration of renewable energy sources into the grid, photovoltaic systems, and electric vehicles.

## Data Availability

The datasets used and/or analyzed during the current study available from the corresponding author on reasonable request.
